# Multi-approach metabolomics analysis and artificial simplified phytocomplexes reveal cultivar-dependent synergy between polyphenols and ascorbic acid in fruits of the sweet cherry (*Prunus avium* L.)

**DOI:** 10.1371/journal.pone.0180889

**Published:** 2017-07-21

**Authors:** Mauro Commisso, Martino Bianconi, Flavia Di Carlo, Stefania Poletti, Alessandra Bulgarini, Francesca Munari, Stefano Negri, Matteo Stocchero, Stefania Ceoldo, Linda Avesani, Michael Assfalg, Gianni Zoccatelli, Flavia Guzzo

**Affiliations:** 1 Department of Biotechnology, University of Verona, Verona, Italy; 2 Department of Biological, Chemical and Pharmaceutical Sciences and Technologies, University of Palermo, Palermo, Italy; 3 Department of Women’s and Children’s Health, University of Padova, Padova, Italy; USDA/ARS, UNITED STATES

## Abstract

Fruits of the sweet cherry (*Prunus avium* L.) accumulate a range of antioxidants that can help to prevent cardiovascular disease, inflammation and cancer. We tested the *in vitro* antioxidant activity of 18 sweet cherry cultivars collected from 12 farms in the protected geographical indication region of Marostica (Vicenza, Italy) during two growing seasons. Multiple targeted and untargeted metabolomics approaches (NMR, LC-MS, HPLC-DAD, HPLC-UV) as well as artificial simplified phytocomplexes representing the cultivars Sandra Tardiva, Sandra and Grace Star were then used to determine whether the total antioxidant activity reflected the additive effects of each compound or resulted from synergistic interactions. We found that the composition of each cultivar depended more on genetic variability than environmental factors. Furthermore, phenolic compounds were the principal source of antioxidant activity and experiments with artificial simplified phytocomplexes indicated strong synergy between the anthocyanins and quercetins/ascorbic acid specifically in the cultivar Sandra Tardiva. Our data therefore indicate that the total antioxidant activity of sweet cherry fruits may originate from cultivar-dependent interactions among different classes of metabolite.

## Introduction

Many environmental and lifestyle factors, as well as the normal process of aging, can trigger an imbalance between antioxidant defence mechanisms and free radical pressure from excess levels of reactive oxygen species and reactive nitrogen species in the body, increasing the risk of cardiovascular disease, inflammation and cancer [1]. Fruits and vegetables help to prevent these diseases when consumed as part of a healthy diet [2,3] and polyphenols play a prominent role in this protective activity [4–6].

Fruits of the sweet cherry (*Prunus avium* L.) are particularly rich in polyphenols (especially flavonoids, anthocyanins and hydroxycinnamic acids) as well as containing high levels of ascorbic acid and potassium [7,8]. Sweet cherry is an important crop, especially in Asia, Europe and North America, which in 2012 produced 910,928, 721,356 and 399,832 tonnes of fruit, respectively [9]. The five largest producers in the same year were Turkey (480,748 tonnes), the United States (384,647 tonnes), Iran (~150,000 tonnes), Italy (104,766 tonnes) and Spain (96,946 tonnes) [9]. In some parts of west Asia, Europe and South America, sweet cherry and other *Prunus* species are the first fresh fruits of the season [10]. In Italy, most sweet cherry orchards are found in Puglia (12,302 ha), followed by Emilia Romagna (2,669 ha) and Veneto (2,567 ha) [11]. In Veneto, Marostica cherries from the Vicenza province were awarded protected geographical indication (PGI) status in 2002 [12]. Many different sweet cherry cultivars are grown, differing in properties such as fruit size, skin colour, pulp colour/consistency, harvest period and disease resistance [13–22].

Both *in vitro* assays and clinical trials have demonstrated the anti-inflammatory properties of sweet cherry metabolites. For example, sweet cherry anthocyanins inhibit the cyclooxygenases COXI and COX II, which are involved in inflammatory responses [23]. Furthermore, the consumption of sweet cherry fruits (280 g/day for 4 weeks) reduces the prevalence of serum biomarkers of inflammation, including C-reactive protein (CRP), ferritin, interleukin-18 (IL-18), tumour necrosis factor alpha (TNFα), interleukin-1 receptor agonist (IL-1Ra), endothelin-1 (ET-1), extracellular newly identified ligand for the receptor for advanced glycation end products (EN-RAGE), and plasminogen activator inhibitor-1 (PAI-1) in healthy adults [24,25]. Sweet cherry consumption could therefore help to reduce the risk of arthritis (indicated by CRP, TNFα, IL-18 and IL-1Ra), cardiovascular disease (CRP, ferritin, ET-1, EN-RAGE, PAI-1 and IL-18), cancer (ET-1), and hypertension (ET-1) [25]. Finally, a single 280-g dose of fresh cherries administrated to healthy women was shown to reduce the levels of serum urate, which can cause gout when deposited in joint tissues [26]. More recently, a large case-crossover observational study showed that the consumption of sweet cherries or corresponding extracts correlates with a lower risk of gout attacks [27]. The studies described above predominantly focused on the Bing cultivar [24–26], which is the most common cultivar produced in the United States, whereas the observational study did not focus on a particular cultivar [27].

The compositional properties of sweet cherry fruits vary substantially among different cultivars and are also influenced by fruit ripening, climatic conditions, soil composition and agricultural practices, resulting in significant differences in the levels of protective secondary metabolites. We therefore compared the metabolomes and antioxidant properties of 18 cultivars of sweet cherry grown in the Marostica PGI zone, using *in vitro* assays and complementary metabolomic platforms (NMR, HPLC-DAD, HPLC-UV, HPLC-ESI-MS), including quantitative targeted analysis as well as untargeted LC-MS analysis. Given the variability of the secondary metabolome in sweet cherry fruits, we sampled in two vintages of each cultivar, and for one cultivar we also sampled fruits from plants grown in a distant geographic zone with distinct pedoclimatic conditions. We also investigated the antioxidant activity of artificial simplified phytocomplexes mimicking the natural phytocomplex composition, because these can be manipulated for remove one or more metabolites allowing us to test for additive and synergistic effects [28].

## Materials and methods

### Plant material, sampling and fruit quality analysis

**Fig 1:**
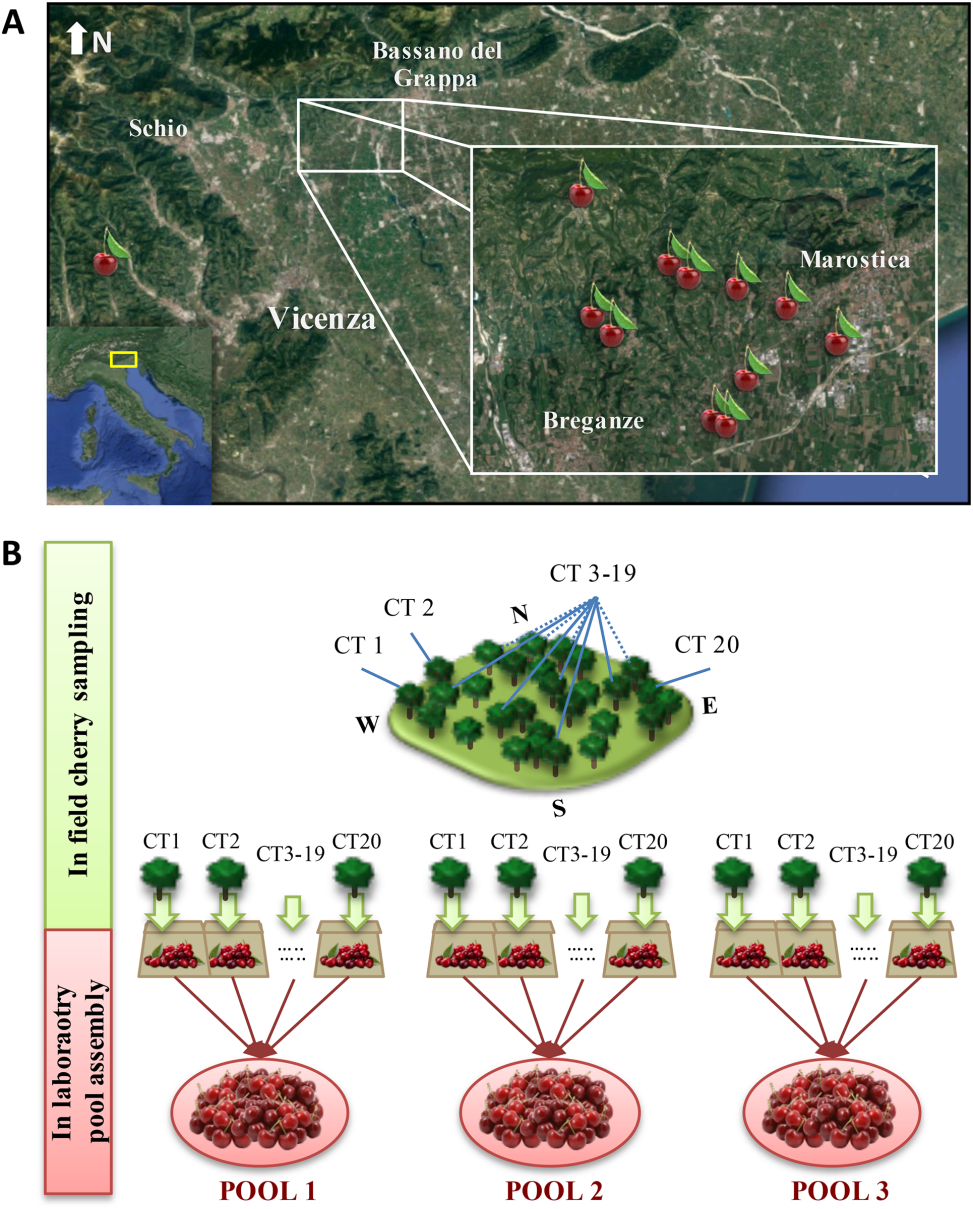
Sweet cherry sampling plan. A) The Vicenza region in Italy, showing the locations of the 12 different farms as cherry icons. B) The two-step sampling plan: field sampling by collecting cherries from 20 trees, and laboratory sampling by assembling three pools of 50 cherries. CT = cherry tree.

Sweet cherries were collected when fully ripe from 18 different cultivars grown on 12 farms in the Marostica PGI zone in Vicenza, during the 2014 and 2015 growing seasons (Fig 1 and S1 Table). About 50 fruits were collected per tree, from up to 20 different trees when available. The fruits were collected from various positions on each tree and from trees of different ages growing at different positions in the orchard. The fruits were placed in labelled paper bags for transfer to the laboratory. On the same day, equal numbers of fruits from the bags representing individual trees were randomly chosen to create pooled samples of 50 fruits. The procedure was carried out three times to generate three biological replicates for metabolomics analysis. Similar procedures were used to assemble three pools of 10 fruits for ascorbic acid analysis, and one pool of 30 cherries for fruit quality analysis. For cultivars grown on different farms, the 50-fruit pools included an equal number of fruits from each orchard.

For metabolomics analysis, the drupes were rapidly deseeded using a manual cherry stoner (Westmark, Lennestadt-Elspe, Germany) and cut into four equal vertical slices, two of which were immediately frozen in liquid nitrogen to generate technical replicates for each of the three biological replicates. One technical replicate was reserved to provide backup material if necessary. All 50 fruit pieces in each pool were powdered in liquid nitrogen using an A11 basic analytical mill (IKA-Werke, Staufen, Germany) and the powder was stored at –80°C.

For fruit quality analysis, the hardness, weight, degrees Brix and pH were determined for 30 fruits. The hardness of each fruit was measured with a sclerometer (Agro Technologies) at four different positions. The fruits were then divided into five groups of six, and each group was pulverized with a mortar and pestle. The pH of the juice was measured using a standard pH meter (micropH 2001, Crison) and the degrees Brix was determined using a DBR 35 digital refractometer (Ningbo Kingstic, Ningbo, China).

### Sample preparation and analysis by NMR spectroscopy

NMR samples were prepared by weighing out and thawing 2–3 g of frozen powder from the three pools of 50 fruits prepared as described above, and then vortexing and sonicating the resulting juice for 15 min in a cold water bath. The raw juice was centrifuged for 10 min at 15,000 *g* and 4°C, and then the supernatant was centrifuged for 20 min at 18,000 *g* and 4°C. We then diluted 0.56 ml of the aqueous extract with buffer to a final volume of 0.7 ml containing 150 mM potassium phosphate, 5% D_2_O (Cambridge Isotope Laboratories, Cambridge, UK), 0.02% NaN_3_ and 1 mM 4,4-dimethyl-4-silapentane-1-sulfonic acid-d6 (DSS-d6) (Sigma–Aldrich, St Louis, MO, USA). The pH of samples was measured with a mini-electrode and adjusted to pH 6.

One-dimensional ^1^H-NOESY spectra were acquired from the aqueous extracts at 25°C using a Bruker Avance III 600 MHz NMR spectrometer (Bruker, Karlsruhe, Germany) equipped with a triple resonance TCI cryogenic probe and operating at a ^1^H Larmor frequency of 600.13 MHz. Data were acquired automatically using IconNMR software and an automatic sample changer (both from Bruker). A time delay of 5 min was set between sample injection and pre-acquisition calibrations to allow for complete temperature equilibration. The experiments were recorded with 64 free induction decays (FIDs), 64,000 data points, a spectral width of 20 ppm, 10 s of recycle delay, and a mixing time of 100 ms. The water signal was suppressed using a solvent saturation scheme.

NMR spectra were processed using Topspin v3.2 (Bruker). FIDs were multiplied by an exponential weighting function with line broadening of 0.3 Hz before Fourier transformation, phasing and baseline correction. Processed spectra were referenced to the DSS-d6 singlet signal. Metabolites were annotated using the Profiler module of Chenomx NMR Suite v8.0 (Chenomx, Alberta, Canada) combined with reference compound spectra deposited in the Biological Magnetic Resonance Bank (http://www.bmrb.wisc.edu/). Quantification was achieved by the integration of NMR signals using the DSS-d6 as an internal standard, but for overlapping signals such as fructose we used the Chenomx software instead. The resulting concentrations were corrected for the dilution factor and converted into milligrams per 100 grams of fresh fruit based on the initial recorded weights and volumes. The performance of the NMR laboratory in terms of fingerprinting and quantitative multicomponent analysis was recently assessed in a large-scale inter-laboratory comparison (quality control parameter for the laboratory, QP << 2.0) [29].

### Metabolite extraction for HPLC-DAD and HPLC-MS analysis

For the analysis of polyphenols, we extracted ~300 mg of frozen powder from each of the pooled samples in 3, 10 or 15 volumes of cold methanol acidified with 1% (v/v) HCl (37% v/v). There was no difference in extraction efficiency with different extraction volumes, so we used three volumes of the solvent for all subsequent experiments. The samples were vortexed for 1 min and sonicated at 40 kHz in an ultrasonic bath (Falc Instruments, Bergamo, Italy) for 15 min and then centrifuged twice for 10 min at 18,000 *g* and 4°C. The supernatant was stored at –20°C. The methanolic extracts were diluted 1:2, 1:3, 1:5 and 1:10 in LC-MS-grade water, passed through 0.2-μm Minisart RC4 filters (Sartorius-Stedim, Göttingen, Germany) and analysed by HPLC-MS and HPLC-DAD. There was no difference in data quality at different dilutions so all further experiments were carried out using the dilution ratio 1:3.

For the analysis of ascorbic acid in fresh fruits, the three 10-fruit pools described above were processed and analysed within a few hours after collection. Each fruit was pitted and 20 g of flesh (~2 g/fruit) was used for ascorbic acid extraction and analysis as previously described [30], with some modifications. Briefly, the flesh was homogenized with 0.2% (w/w) potassium metabisulfite powder with a T25 Ultra Turrax homogenizer (IKA-Werke) for 30 s at 24,000 rpm on ice. We then diluted 1 g of the homogenized sample with 20 mM NaH_2_PO_4_ (pH 2.14) to a final volume of 10 ml. The solution was vortexed twice for 30 s and centrifuged for 5 min at 9,200 g and 4°C. The supernatant was passed through a 0.2-μm Minisart RC4 filter and stored at 4°C in the dark. For the analysis of ascorbic acid in frozen fruit powder, ~150 mg of powder from the three 50-fruit pools prepared as described above was extracted in nine volumes of 0.02% (w/v) potassium metabisulfite in 20 mM sodium dihydrogen phosphate buffer (pH 2.14). The extract was then processed as described above for the fresh fruits.

### HPLC-DAD analysis

The quantitative analysis of polyphenols by HPLC-DAD was carried out by injecting 30 μl of the acidified methanolic extract in to a Gold 126 Solvent Module coupled with a Gold 168 Diode Array Detector and a System Gold 508 Autosampler (all provided by Beckman Coulter, Fullerton, CA, USA). The samples were eluted in a gradient of two solvents, namely 0.5% (v/v) formic acid plus 5% (v/v) acetonitrile in LC-MS-grade water (solvent A) and 100% acetonitrile (solvent B), using the following 45-min program: from 0 to 10% B in 2 min, from 10 to 20% B in 10 min, from 20 to 25% B in 2 min, from 25 to 70% B in 7 min, isocratic elution at 70% B for 5 min, from 70% to 90% B in 1 min, isocratic elution at 90% B for 4 min, and from 90% B to 0% B in 1 min. The column was then equilibrated for 18 min in 100% A. The metabolites were separated by passing them through a C18 guard column (7 x 2.1 mm) and an analytical Alltima HP C18 column (150 x 2.1 mm, particle size 3 μm) both from Alltech Associates (Derfield, IL, USA) at a flow rate of 0.2 ml/min. The data were collected using 32 Karat Workstation v7.0 (Beckman Coulter). Calibration curves were established using serial dilutions of the authentic standards chlorogenic acid, p-coumaric acid and cyanidin 3-O-glucoside (kuromanin) all from Extrasynthese (Genay, France) and quercetin (Sigma–Aldrich). The following wavelengths were chosen for metabolite quantification: chlorogenic acid and p-coumaric acid = 320 nm, quercetin = 350 nm, and cyanidin 3-O-glucoside = 520 nm. The chlorogenic and neochlorogenic acids were quantified as chlorogenic acid equivalents, p-coumaroyl quinic acid was quantified as p-courmaric acid equivalents, quercetin 3-glucoside and quercetin 3-rutinoside were quantified as quercetin equivalents, and the anthocyanins were quantified as cyanidin-3-O-glucoside equivalents.

The quantitative analysis of ascorbic acid by HPLC-DAD was carried out using the same equipment as described above, but samples with an injection volume of 10 μl were eluted under isocratic conditions with 20 mM sodium phosphate monobasic buffer (pH 2.14) for 20 min at room temperature at flow rate of 0.2 ml/min. A calibration curve was established with serial dilutions of L-ascorbic acid authentic standard and a wavelength of 420 nm was used for quantification.

### Untargeted HPLC-MS analysis

Untargeted HPLC-MS analysis was carried out using a Gold 127 HPLC system equipped with a refrigerated System Gold 508 Autosampler (both supplied by Beckman Coulter) using the same column and elution method described above for the HPLC-DAD analysis of polyphenols. The HPLC system was coupled on-line with an Esquire 6000 electrospray ionization (ESI) ion trap mass spectrometer (Bruker). Instrument performance was monitored using two quality control (QC) samples. QC1 was a mixture of six authentic commercial standards representing molecules with diverse retention times: 0.1 mg/ml α-resorcylic acid, 0.02 mg/ml chlorogenic acid, 0.1 mg/ml daidzein, 0.070 mg/ml kaempferol-3-O-rutinoside, 0.020 mg/ml resveratrol and 0.01 mg/ml sakuranetin dissolved in methanol and diluted 1:2 (v/v) in LC-MS-grade water. QC2 was a mixture of equal parts of frozen powder from all the cherry cultivars, extracted as described above for polyphenol analysis, and diluted 1:3 (v/v) with LC-MS-grade water. The 20-μl samples were analysed in groups of 11, including one QC1 and one QC2 sample at the beginning of the group analysis, followed by a cycle of machine cleaning. The samples were analysed in alternating mode, with a scan range of 50–1500 Da and a target mass of 400 *m/z*. Data were recorded using Esquire Control v5.2 and processed with Data Analysis v3.2 (both from Bruker). Further data for annotation were acquired in MS/MS and MS^3^ modes. The amplitude of fragmentation was set to 1 V. Nitrogen was used as the nebulizing and drying gas. The ion source parameters were: 50 psi at 350°C for the nebulizing gas and 10 L/min for the drying gas. Helium was used for collision induced dissociation.

The retention times, UV-visible absorption curve, *m/z* values, and fragmentation trees (MS/MS and MS^3^) were used to identify the metabolites based on an in-house library of mass and UV/VIS spectra representing authentic commercial standards. When this was not sufficient, *m/z* values and fragmentation patterns were compared with those found in MassBank (www.massbank.jp) and in the scientific literature. Neutral losses of 132, 146 and 162 Da were considered to represent the loss of pentose, deoxyhexose and hexose sugars, respectively. The fragments of *m/z* 179 (MS/MS) → 135 (MS^3^), 163→119, 191→173, and 193→134,149,178 were used to annotate caffeic, coumaric, quinic and ferulic acid derivatives.

### LC-MS data extraction, alignment and data analysis

Raw.d LC-MS data files were converted to.cdf files and automated *m/z* feature detection, deconvolution and alignment was carried out using MZmine v2 (http://mzmine.github.io/). Median fold change normalization was applied and the resulting dataset was log-transformed and mean centred prior to statistical analysis. Exploratory principal component analysis (PCA) was applied to highlight cluster structures related to the cultivars, outliers and patterns due to the two growing seasons.

The relationship between metabolite profiles and the outcomes of the *in vitro* assays were modelled by projection to latent structures regression based on variable influence on projection selection (VIP-based PLS) [31] and simple linear regression. The former technique allowed the evaluation of synergistic effects among the recorded variables from a multivariate perspective, whereas the latter independently quantified the effect of each variable on the observed outcomes. A stability selection procedure using VIP-based PLS was implemented to highlight the most relevant subset of variables for the explanation of the outcomes. Specifically, 1000 random subsamples of the collected samples were extracted by Monte-Carlo sampling (with a prior probability of 0.70), and then VIP-based PLS was applied to each subsample to yield a set of 1000 regression models. The relevant variables were identified as those selected in more than 50% of all models. The predictive performance of each model was estimated as a standard deviation error in prediction (SDEP) by predicting which samples would be excluded during Monte-Carlo sampling. The VIP threshold for the VIP-based PLS was determined by maximizing the Q^2^ calculated by seven-fold full cross-validation. A permutation test was applied to the response to avoid over-fitting. A similar strategy was implemented for simple linear regression.

PCA was carried out using SIMCA v14.0 (MKS Data Analytics Solutions, Umeå, Sweden) whereas VIP-based PLS, simple linear regression and stability selection were implemented in R v3.1.2 (R Foundation for Statistical Computing, Vienna, Austria).

### *In vitro* antioxidant assays

Antioxidant activity was determined *in vitro* using the ferric reducing antioxidant power (FRAP) assay [32,33] and the 2,2-azino-*bis*(3-ethylbenzothiazoline-6-sulfonic acid) (ABTS) assay [34], both with some modifications. About 150 mg of powder, prepared as described above, was mixed for 30 s with nine volumes of methanol acidified with 1% (v/v) HCl (37%) and centrifuged for 10 min at 10,621 *g* and 4°C. The supernatants were diluted with four volumes (FRAP assay) or one volume (ABTS assay) of acidified methanol and stored at –20°C. The samples were analysed in 96-well flat-bottom microtest plates (Sarstedt, Nümbrecht, Germany) using a Power Wave microplate reader and Gene5 v1.08 software (both from Bio-Tek, Winooski, VT, USA).

Artificial simplified phytocomplexes were designed according to the metabolic composition of the cultivars Sandra Tardiva, Sandra and Grace Star harvested in 2015. Because ascorbic acid is unstable, its concentration in the frozen powder was determined just before the antioxidant activity assay. The following commercial standards were used: neochlorogenic acid, chlorogenic acid, quercetin-3-O-glucoside, cyanidin-3-O-rutinoside and cyanidin-3-O-glucoside (Extrashynthese), quercetin-3-O-rutinoside and L-ascorbic acid (Sigma–Aldrich). The exact composition of each phytocomplex is summarized in S2 Table. To determine the antioxidant proprieties of the phytocomplexes, every mix was prepared in methanol acidified with 1% (v/v) HCl (37%), and diluted for analysis using the FRAP assay.

The FRAP assay reaction mixture was prepared by mixing 300 mM aqueous sodium acetate buffer (pH 3.6), 10 mM 2,4,6-tripyridyl-s-triazine (TPTZ) in 40 mM HCl, and 20 mM aqueous FeCl_3_ at a ratio of 10:1:1. The assay mixture was divided into three 200-μl aliquots and each aliquot was mixed with 20 μl of cherry extracts or phytocomplexes in a 96-well microplate. After incubation at 37°C for 15 min, the samples were allowed to cool to room temperature for 4 min and the absorbance was recorded at 593 nm. Values acquired were compared with a standard curve established by preparing serial dilutions of 6-hydroxy-2,5,7,8-tetramethylchroman-2-carboxylic acid (Trolox) in methanol. The results were expressed as mmol Trolox equivalents (TE)/g of cherry powder or phytocomplex. Each experiment was carried out twice.

The ABTS assay mixture was prepared by mixing 7 mM ABTS in water with an equal volume of 2.45 mM K_2_O_8_S_2_ and stirring in the dark at room temperature for 12 h. The mixture was then diluted in methanol until the absorbance at 734 nm was 0.700 (±0.02). The assay mixture was divided into three 200-μl aliquots and each aliquot was mixed with 20 μl of cherry extracts in a 96-well microplate. The reaction was allowed to proceed for 1 h in the dark at 30°C and then the absorbance was recorded at 734 nm. The absorbance values were compared with a standard Trolox curve as described above for the FRAP assay and the results were likewise expressed as mmol Trolox equivalents (TE)/kg cherry powder.

## Results

### Sampling plan

Mature fruits representing 18 sweet cherry cultivars were collected during the 2014 and 2015 growing seasons from 12 farms in the Marostica GPI area (Fig 1). Representative sampling was ensured by collecting fruits of the same cultivar from as many different orchards as possible, and by surveying each orchard so that fruits were collected from different types of tree in terms of age, exposure and elevation. We selected healthy fruits randomly from different positions in each tree when they were ripe and ready for market based on the expertise of the growers. Fruits from each tree were collected in separate paper bags. In the laboratory we assembled three independent pools of 50 fruits equally representing different trees and orchards. Fruit quality was assessed by measuring hardness, pH and degrees Brix, and we found that the maturity was similar for each cultivar and vintage (S1 Table).

Within each cultivar, the three independent pools of fruits showed substantially overlapping chromatographic profiles, indicating that 50 fruits were sufficient for representative analysis. Furthermore, the profiles of each cultivar were similar across the two growing seasons, whereas the profiles of different cultivars showed substantial differences. The three cultivars Early Bigi, Burlat and Sandra Tardiva are shown as representative examples in Figure A in S1 File. For the Early Bigi cultivar in the 2015 growing season, we also sampled fruits from a distant orchard in Sicily featuring very different pedoclimatic conditions. Despite the substantial environmental differences between Sicily and the Marostica PGI zone in Vicenza, the metabolic profiles of the samples were similar. The PCA model generated from the metabolites detected by HPLC-ESI-MS clustered all the Early Bigi samples (2014, 2015 and Sicily-2015) and separated them from the other cultivars, suggesting that metabolic differences between the sweet cherry cultivars predominantly reflected their underlying genetic diversity (Figure A in S1 File).

### Quantitative analysis of the sweet cherry metabolome

The main primary and secondary metabolites (including ascorbic acid) in the different cultivars were quantified by NMR spectroscopy and HPLC-DAD (S3 Table). The NMR data revealed the levels of some sugars (glucose, fructose and xylose), organic acids (fumaric and malic acids) and free amino acids, whereas HPLC-DAD allowed us to measure the levels of anthocyanins (cyanidins), flavonols (quercetins) and hydroxycinnamic acids, neochlorogenic acid, chlorogenic acid and p-coumaroyl quinic acid, as well as ascorbic acid.

**Fig 2:**
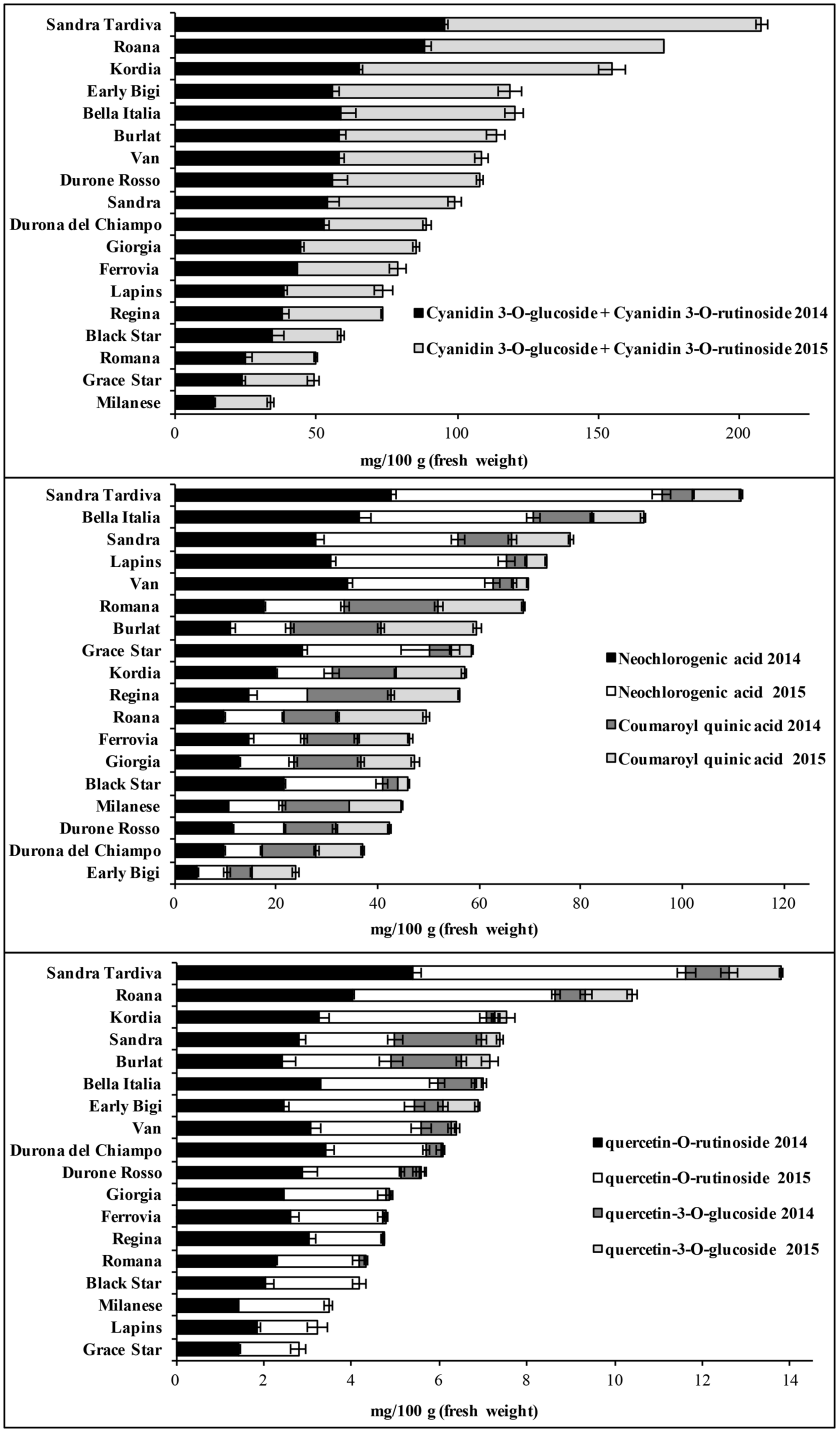
Polyphenol profiles of fruits representing 18 sweet cherry cultivars (2014 and 2015 vintages). Values represent the levels (in mg per 100 g fresh weight) of cyanidin 3-O-glucoside and 3-O-rutinoside, neochlorogenic and coumaroyl quinic acids, quercetin 3-O-glucoside and 3-O-rutinoside, and the levels of each specific metabolite. Data are means ± standard deviations (n = 3).

Glucose and fructose were the most abundant sugars and there were no significant differences in sugar content between the two growing seasons, with the exception of the Lapins and Black Star cultivars, in which the sugar content was significantly lower in 2015 (p < 0.05). Malic acid was the most abundant organic acid and levels were significantly higher in 2015 in all the cultivars except Lapins and Black Star, which contained significantly lower levels than other cultivars (p < 0.05). The ascorbic acid content was 7–15 mg/100 g and varied between the two growing seasons (S3 Table). The main polyphenolic components in the fruits were anthocyanins (up to 114 mg/100 g), hydroxycinnamic acid (up to 65 mg/100 g) and flavonoids (up to 7 mg/100 g). There were substantial differences in polyphenol levels among the cultivars (Fig 2). For example, the anthocyanin content ranged from 14 mg/100 g in Milanese fruits during 2014 to 114 mg/100 g in Sandra Tardiva fruits during 2015.

The PCA model for polyphenolic secondary metabolites showed a strong varietal fingerprint, i.e. samples representing the same variety clustered together closely (Figure B1 in S1 File). The PCA loadings showed that cyanidins and neochlorogenic acid were strongly involved in this varietal clustering (Figure B2 in S1 File). In contrast, the PCA model for primary metabolites (sugars, organic acids and amino acids) showed a much weaker varietal fingerprint (Figure B3 in S1 File). Both PCA models also showed a weak vintage effect, which was stronger for the primary metabolites. In the score scatter plot of the model for primary metabolites, clusters corresponding to the different vintages could be distinguished for some cultivars (Black Star, Lapins, Early Bigi, Roana, Kordia, Van, Sandra Tardiva, Durona del Chiampo and Durone Rosso), whereas the effect for secondary metabolites was weaker, with only cultivars Sandra Tardiva and Kordia clustering according to the vintage. The primary metabolites glucose, fructose, malic acid and arginine appeared to be the most dependent on vintage (Figure B4 in S1 File) and among the secondary metabolites the same applied to the cyanidins, neochlorogenic acid and coumaroyl quinic acids (Figure B5 in S1 File). Fructose and glucose accumulated to higher levels in some varieties during 2014 (Black Star and Lapins) but in others during 2015 (Roana, Durona del Chiampo and Sandra Tardiva). Because sugars accumulate during ripening, the observed differences in sugar levels may reflect slight variations in the ripening stage during sampling. In the Sandra Tardiva cultivar, the levels of all the secondary metabolites we measured were higher in 2015 than 2014, whereas the Kordia cultivar accumulated higher levels of cyanidins but lower levels of neochlorogenic acid in 2015.

### Untargeted-LC-MS analysis

HPLC-ESI-MS analysis allowed the detection of many more metabolite than HPLC-DAD or NMR spectroscopy because the platform is more sensitive (S4 Table) but the matrix effect and ion suppression/enhancement prevent absolute quantitative analysis. However, both HPLC-ESI-MS and HPLC-DAD produced similar quantitative results for the most abundant metabolites that can be detected using both techniques, suggesting that HPLC-ESI-MS data are suitable at least for relative quantitation, i.e. comparison between samples (Figure C in S1 File). In addition to the metabolites already detected by HPLC-DAD (and their isotopes and adducts), HPLC-ESI-MS analysis revealed the presence of the flavan-3-ols catechin and epicatechin, plus various procyanidins, flavonoids and hydroxycinnamic acids, and the anthocyanins pelargonidin rutinoside and peonidin rutinoside (S4 Table and Figure D in S1 File). The dataset comprised 203 *m/z* features, representing 38 different putatively annotated metabolites, their isotopes and adducts, and 104 unidentified signals.

**Fig 3:**
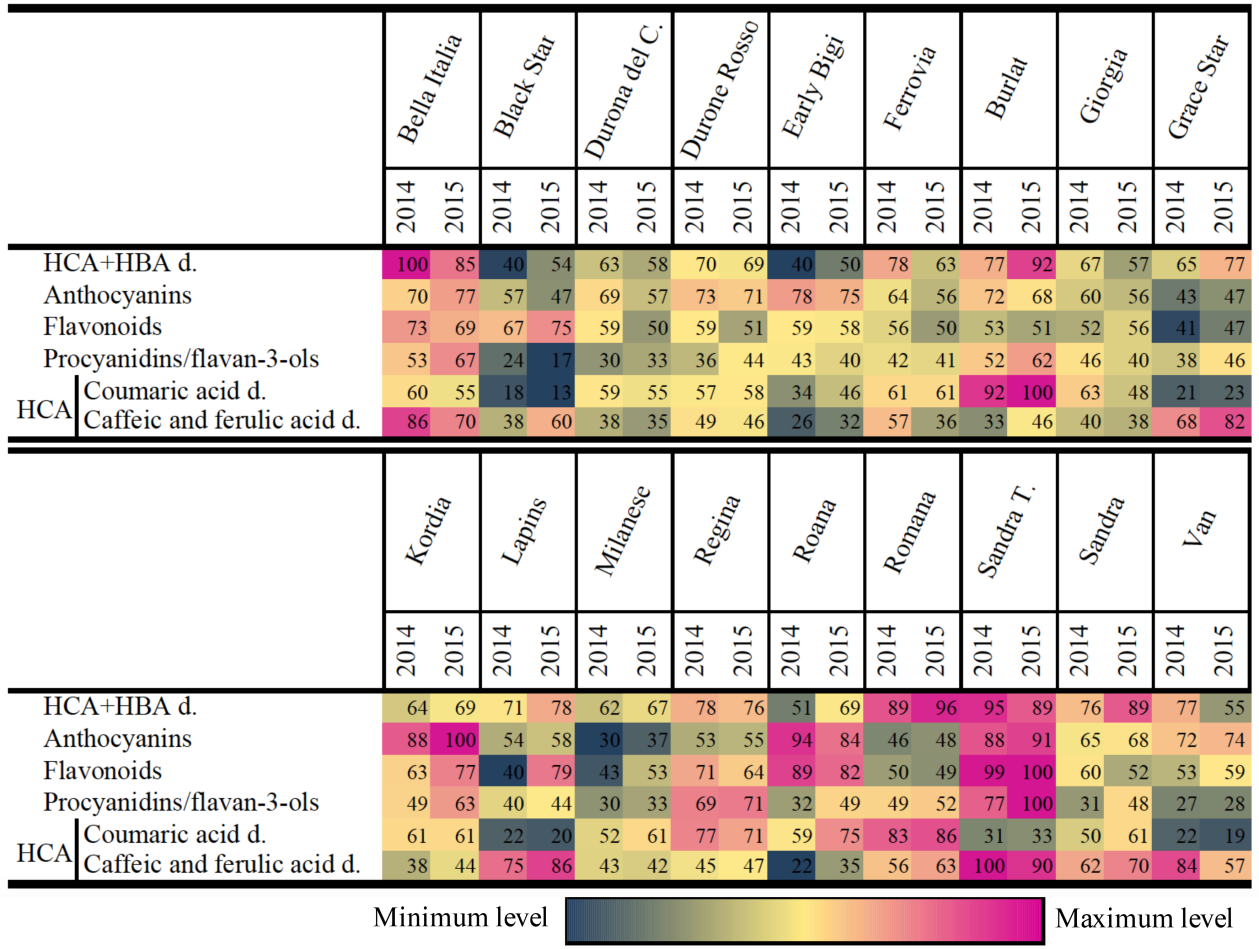
Heat map of the principal classes of metabolites that accumulate in each cultivar. The data show the proportion of each metabolite class (percentage) and the year trend in the different cultivars. The colour gradient ranges from blue (low abundance) to red (high abundance). Legend: hydroxycinnamic acids, HCAs; hydroxybenzoic acids, HBAs; derivatives, d.; Durona del Chiampo, Durona del C; Sandra Tardiva, Sandra T.

Exploratory PCA did not highlight outliers and produced a sample representation comprising four principal components explaining 68.3% of the total variance, with samples clustered by cultivar (Figures E1-2 in S1 File). The composition of the metabolome is presented as a relative quantitation heat map in Fig 3 (showing classes of metabolites) and Figure D in S1 File (showing individual metabolites with putative annotations). The cultivar with the highest polyphenol content was Sandra Tardiva, followed by Bella Italia, Kordia, Roana, Regina and Burlat. The cultivar with the lowest polyphenol content was Milanese, followed by Black Star, Grace Star, Durona del Chiampo and Giorgia. The remaining cultivars showed intermediate levels. Interestingly, among the hydroxycinnamic acid derivatives, some of the cultivars showed an inverse relationship between the abundance of coumaric and caffeic-ferulic acid derivatives: Burlat fruits accumulated high levels of coumaric acid derivatives but low levels of caffeic/ferulic acid derivative, whereas the opposite profile was typical for cultivars Grace Star, Lapins, Sandra Tardiva and Van. The relative metabolite levels in each of the cultivars were similar across the two growing seasons.

### *In vitro* antioxidant activity of sweet cherry phytocomplexes and corresponding artificial simplified phytocomplexes

**Fig 4:**
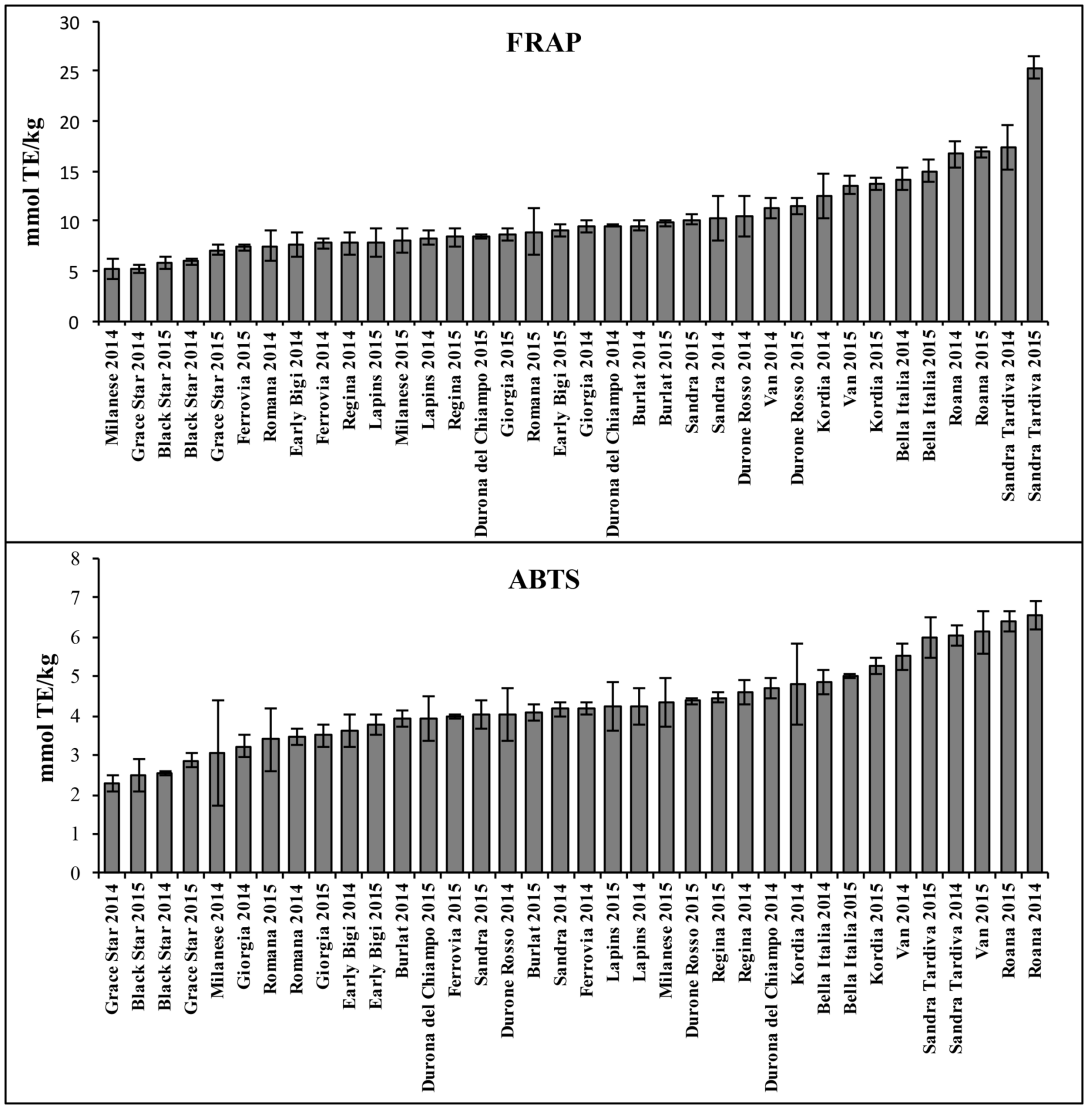
Antioxidant properties of sweet cherry fruit extracts from different cultivars. The results of the FRAP and ABTS assays are expressed as mmol of Trolox equivalents per kg fresh weight. The values are means ± standard deviations (n = 3).

ABTS and FRAP assays were carried out to characterize the *in vitro* antioxidant properties of fruit extracts from each cultivar, with the values expressed in terms of Trolox equivalent antioxidant capacity (TEAC) as shown in Fig 4. The FRAP results were expressed as log_10_(FRAP). The two assays gave similar albeit not identical results (Pearson correlation coefficient = 0.87). Cultivars Sandra Tardiva, Roana, Bella Italia, Kordia and Van showed the highest antioxidant activity and cultivars Grace Star, Black Star and Milanese showed the lowest antioxidant activity. There was no significant vintage effect in any of the cultivars (p > 0.20).

Potential relationships between the antioxidant activities determined *in vitro* and the metabolite profiles of each cultivar were investigated using both the accurate quantitative data for seven metabolites (targeted HPLC-DAD analysis) and the broader but less accurate dataset produced by untargeted LC-MS analysis. For the HPLC-DAD dataset, the PLS model for the ABTS assay comprised A = 2 components, a correlation coefficient (R^2^) of 0.61 and a standard deviation error calculated by seven-fold full cross-validation (SDECV) of 0.9. Similarly, the PLS model for the FRAP assay comprised A = 2 components, with R^2^ = 0.77 and SDECV = 0.09 for log_10_(FRAP). The predictive components of the models are plotted against the results of the two assays in Figures F1-2 in S1 File. Stability selection produced a set of models showing mean SDEPs of 0.8 and 0.09 for the ABTS and log_10_(FAP) data, respectively. We selected 2-caffeoyl quinic acid, cyanidin-3-O-glucoside + cyanidin-3-O-rutinoside, peonidin (coumaroyl) rutinoside and quercetin-O-rutinoside as the most relevant variables to explain the ABTS results, whereas 1-caffeoyl quinic acid, coumaroyl quinic acid, 2-caffeoyl quinic acid, cyanidin-3-O-glucoside + cyanidin-3-O-rutinoside, peonidin (coumaroyl) rutinoside, quercetin-O-rutinoside and quercetin-3-O-glucoside were selected as the most important variables to explain the log_10_(FRAP) results. Simple linear regression modelling allowed us to identify some metabolites that individually describe the ABTS and log_10_(FRAP) results. Specifically, we selected cyanidin-3-O-glucoside + cyanidin-3-O-rutinoside (R^2^ = 0.57, mean SDEP = 0.7, mean p = 8 x 10^−5^ for ABTS; R^2^ = 0.71, mean SDEP = 0.08, mean p = 1 x 10^−5^ for log_10_(FRAP)) and quercetin-O-rutinoside (R^2^ = 0.54, mean SDEP = 0.8, mean p = 1 x 10^−4^ for ABTS; R^2^ = 0.66, mean SDEP = 0.09, mean p = 5 x 10^−5^ for log_10_(FRAP)).

PLS modelling of the untargeted metabolomics dataset produced a model with A = 2 components for the ABTS data (R^2^ = 0.65 and SDECV = 0.9) and a model with A = 2 components for the log_10_(FRAP) data (R^2^ = 0.82 and SDECV = 0.09). The predictive components of the models are plotted against the results of the two assays in Figures F3-4 in S1 File. Stability selection produced a set of models showing mean SDEPs of 0.9 and 0.1 for the ABTS and log_10_(FAP) data, respectively. S5 Table lists the relevant metabolites highlighted by stability selection. The three metabolites selected for the ABTS assay were also relevant for the FRAP assay. The simple linear regression model is summarized in S6 Table.

**Fig 5:**
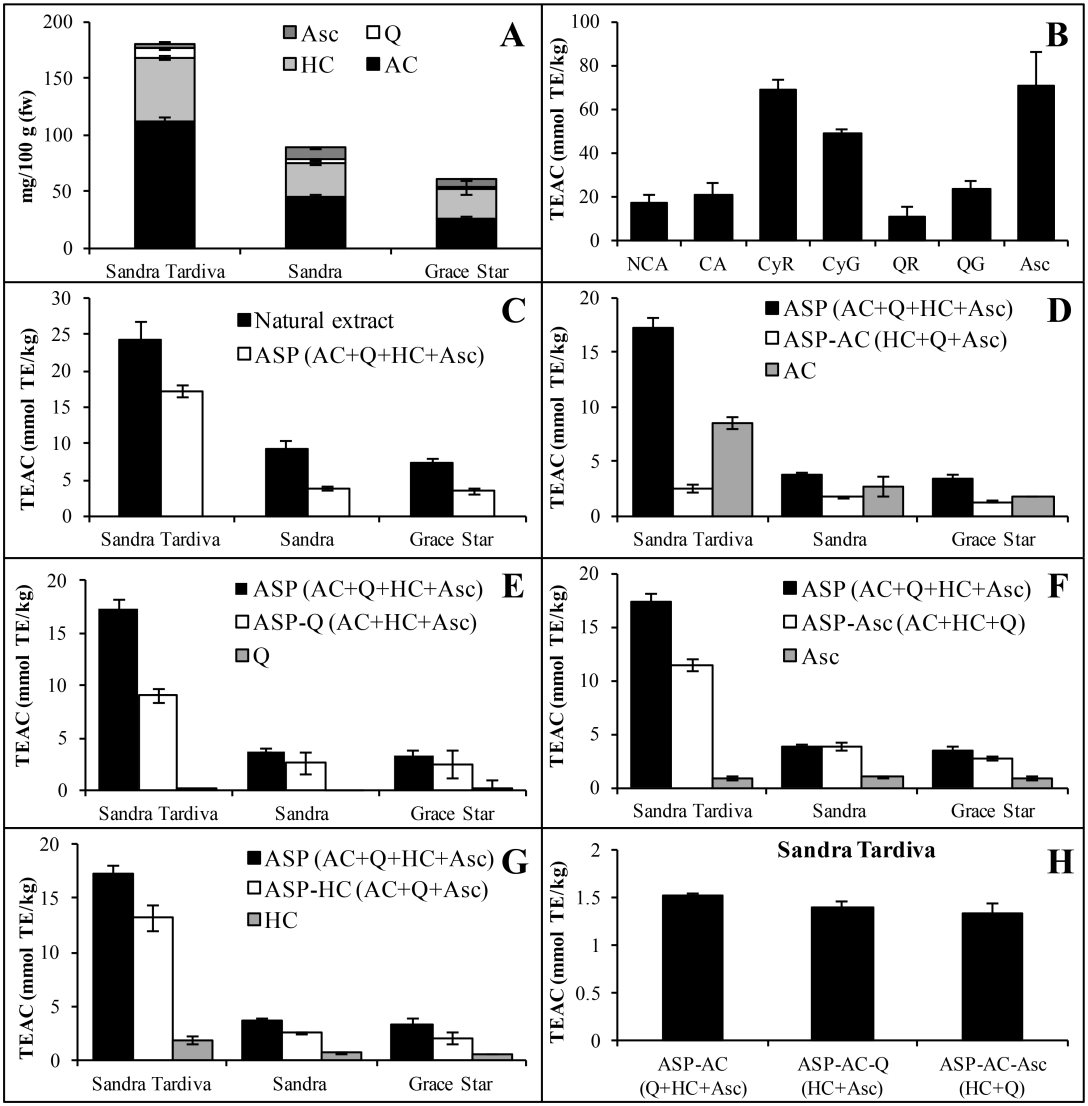
Trolox equivalent antioxidant capacity (TEAC) of individual metabolites and artificial simplified phytocomplexes (ASPs). A) Natural content (mg per 100 g fresh weight (fw) of fruit) of the principal metabolites that accumulate in the three selected cultivars. Black = anthocyanin (AC) content, light grey = hydroxycinnamic acid (HC) content, white = quercetin content, dark grey = ascorbic acid (Asc). B) TEAC of specific metabolites expressed as mmol of Trolox equivalents per kg fresh fruit. Abbreviations: neochlorogenic acid, NCA; chlorogenic acid, CA; cyanidin 3-O-rutinoside; CyR; cyanidin 3-O-glucoside, CyG; quercetin 3-O-rutinoside, QR; Quercetin 3-O-glucoside, QG; Ascorbic acid, Asc. C) TEAC of natural extracts and artificial simplified phytocomplexes of the three selected cultivars. The detailed compositions are shown in S2 Table. Abbreviations: anthocyanins, AC; quercetin, Q; hydroxycinnamic acid, HC; Ascorbic acid, Asc. D-G) TEAC of the different artificial simplified phytocomplexes deprived of a specific component. Columns indicate the TEAC of the intact phytocomplexes (black), phytocomplexes deprived of a specific component (white) and the component itself (grey). H) TEAC of three different artificial simplified phytocomplexes resembling the Sandra Tardiva composition without anthocyanins. Data in panels A-H are means ± standard deviations (n = 3).

Both the targeted and untargeted metabolomics datasets reveal that *in vitro* antioxidant activity correlates mainly with the anthocyanin and quercetin content. The possible role of anthocyanins is unsurprising because these well-known antioxidants are abundant in sweet cherry extracts, but quercetins are less abundant than other metabolites such as neochlorogenic acid, so it is unclear whether the correlation between quercetins and *in vitro* antioxidant activity reflects the genuine functional properties of quercetins or a correlation between quercetin and anthocyanin levels, given that both metabolites are produced by related biosynthesis pathways. We therefore selected three cultivars with low (Grace Star), medium (Sandra) and high (Sandra Tardiva) antioxidant activities for the preparation of artificial simplified phytocomplexes, i.e. simplified metabolomes reconstituted *in vitro* using available authentic standards (Fig 5A). Because sweet cherry contains low but not insignificant levels of the antioxidant ascorbic acid, this molecule was also included in the analysis.

First, the overall *in vitro* antioxidant activity of each individual metabolite in the artificial simplified phytocomplexes was measured using the FRAP assay, revealing that cyanidin-3-O-rutinoside shows the highest antioxidant activity *in vitro*, followed by ascorbic acid and cyanidin-3-O-glucoside, whereas the other metabolites have much lower activity (Fig 5B). The antioxidant properties of the artificial simplified phytocomplexes representing the Sandra Tardiva, Sandra and Grace Star metabolomes were then compared to the natural extracts, revealing that that artificial simplified phytocomplexes accounted for 76%, 43% and 40% of the antioxidant activities of cultivars Sandra Tardiva, Sandra and Grace Star, respectively (Fig 5C).

The artificial simplified phytocomplexes were used to study the specific contribution of each class of metabolite to the overall *in vitro* antioxidant activity by eliminating each class in turn and comparing the depleted complexes to the complete complexes and natural extracts (Fig 5D–5G). This strategy allowed us to determine whether the total observed antioxidant activity was additive (in which case the loss of activity in the depleted complex should be equivalent to the activity of the isolated metabolite) or synergistic (in which case the loss should be greater than activity of the isolated metabolite). These experiments clearly showed that the removal of anthocyanins (Fig 5D), quercetins (Fig 5E) or ascorbic acid (Fig 5F) from the Sandra Tardiva artificial simplified phytocomplex resulted in the loss of much more antioxidant activity than could be attributed to the same metabolites in isolation. This result therefore shows that the anthocyanins, quercetins and ascorbic acid act synergistically rather than additively in the Sandra Tardivaartificial phytocomplex. In contrast, the removal of hydroxycinnamic acids reduced the antioxidant activity of the complex to the extent of the activity provided by the same metabolites in isolation, indicating that these compounds have an additive effect in the Sandra Tardiva artificial simplified phytocomplexes (Fig 5G). Interestingly, all the metabolite classes we investigated (anthocyanins, quercetins, hydroxycinnamic acids and ascorbic acid) showed only additive effects in the Sandra and Grace Star artificial simplified phytocomplexes, suggesting the possible existence of cultivar-dependent synergistic activity (Fig 5D–5G).

Given that the three cultivars we tested differed mainly in terms of the anthocyanin content, we conducted an addition experiment to test the activity of Sandra Tardiva artificial simplified phytocomplexes lacking anthocyanins. When these minimal artificial simplified phytocomplexes were also depleted for quercetins or ascorbic acid, the synergistic effects observed in the previous experiment were no longer present, suggesting that the synergy we observed is dependent on the presence of anthocyanins (Fig 5H).

## Discussion

### The phenolic content of sweet cherry is primarily determined by the genetic background

The composition of mature sweet cherry fruits varies greatly according to genotype, environment, climatic factors and agronomic practices [35–39]. We investigated the metabolomic composition of sweet cherry fruits representing 18 cultivars grown in the Italian province of Vicenza to determine the relative impact of these different factors. The sampling plan was designed to ensure that the measurements were reproducible. For each cultivar and vintage, we assembled three replicate pools of 50 fruits with equivalent coverage of different orchards, trees and positions on trees to rule out environmental variations, and we instrumentally verified the ripening stage to rule out metabolic variations during fruit development and ripening. For one cultivar, we also sampled a distant orchard in Sicily to measure the effect of unrelated geographic and pedoclimatic conditions.

Beside slight differences due to the orchards and the year, that are not discussed in this paper, a specific phenolic composition for each of the cultivars, overall not identical but very similar in two different season and also, for one cultivar, in two different the geographic zone, was clearly shown.

The sampling plan was therefore suitable to determine the varietal composition of the fruits, at least for the phenolic components.

Although the 18 cultivars we investigated were metabolically well-separated regardless of orchard and vintage (and in one case also regardless of the geographic zone), previous studies have shown that the environment can have a significant impact on secondary metabolism. For example, the Black Star cultivar in the current study contained only half the cyanidin content reported in a previous investigation, whereas the Ferrovia cultivar contained double the cyanidin content previously reported and our results for the Grace Star cultivar were comparable with the earlier report [40]. Similarly, the Kordia and Regina cultivars in the current study contained double the previously reported levels of cyanidin and hydroxycinnamic acids [41]. The Burlat and Van cultivars have been the subject of extensive metabolic analysis [34, 40, 42–45]. In the Burlat cultivar, these earlier investigators reported neochlorogenic acid levels of 7–64 mg per 100 g fresh fruit (compared to 11 mg in the current study), chlorogenic acid levels of 1–4 mg (2 mg in this study), coumaroyl quinic acid levels of 6–26 mg (1 mg in this study), and cyanidin levels of 10–82 mg (58 mg in this study). In the Van cultivar, the earlier investigators reported neochlorogenic acid levels of 17–87 mg (31 mg in this study), chlorogenic acid levels of 0.25–6 mg (2 mg in this study), coumaroyl quinic acid levels of 4–17 mg (3.5 mg in this study), and cyanidin levels of 14.5–142 mg (54 mg in this study). This variability probably reflects a combination of factors, including the vintage and the environment, even though our data indicate that genotype has the most significant impact on the secondary metabolome. Different extraction methods and buffers could also affect the results. We used a parsimonious method with only one extraction step for the phenolic compounds to avoid metabolite degradation, but this could underestimate the content of some compounds.

To our knowledge only one previous study has characterized the sweet cherry metabolome over two consecutive growing seasons, and the phenolic profile showed vintage effects in most of the cultivars, including the Van cultivar described herein [42]. Furthermore, cultivar and vintage dependent colour index variations have been reported for seven different cultivars, including the Burlat cultivar described herein [46], and for three sour cherry cultivars [47]. Our experiments revealed a strong cultivar-dependent effect on the secondary metabolome regardless of the vintage in 18 cultivars, which agrees with an earlier study based on three local sweet cherry varieties [48]. Unfortunately, detailed sampling methods are often omitted in the reports discussed above, making it difficult to compare the strengths and weaknesses of the different procedures and their impact on the reported metabolic variations.

### The *in vitro* antioxidant activity of sweet cherry primarily reflects the anthocyanin content

Many sweet cherry metabolites possess antioxidant activity, including phenolic compounds and ascorbic acid (included in this investigation) as well as tocopherols [49] and carotenoids [50]. All the phenolic compounds detected in sweet cherry, including anthocyanins, flavonols, hydroxycinnamic acids, flavan-3-ols and procyanidins, have antioxidant activities. This investigation was limited to metabolites with high to medium polarity.

Our preliminary results revealed a strong relationship between the antioxidant activity of sweet cherry extracts and the content of cyanidins and quercetins, and a weaker relationship with the content of hydroxycinnamic acids. The suggested strong impact of quercetins was surprising because sweet cherry fruits contain low levels of these compounds compared to anthocyanins and hydroxycinnamic acids, whereas the weak impact of hydroxycinnamic acids was surprising given their relative abundance. This was even more surprising when the individual *in vitro* antioxidant activity of the main cherry components was measured at constant concentration, showing that the anthocyanins alone provided strong antioxidant activity, whereas the quercetins and hydroxycinnamic acids provided similar and lower levels of activity.

Previous studies have not yielded a consistent picture of the roles of different antioxidant compounds in sweet cherries. Agreeing with our results, a strong correlation was found between the total phenolic content and antioxidant activity of four sweet cherry cultivars (including Van) using the ABTS and 2,2-diphenyl-1-picrylhydrazyl (DPPH) assays, with anthocyanins playing a predominant role [34]. Similarly, ABTS, DPPH and FRAP assays were used to confirm a correlation between the antioxidant properties of sweet cherries, their red colour and the anthocyanin content [51]. In contrast, oxygen radical absorbance capacity (ORAC) assays showed a much weaker correlation between the antioxidant activities and anthocyanin/phenolic content of 24 sweet cherry varieties, with some displaying high antioxidant activity despite a low total phenolic content [40]. Others have reported a significant correlation between antioxidant activity and the levels of p-coumaroyl acid and epicatechin [43] or catechin [52], or a weak correlation between antioxidant activity and total polyphenol/flavonoid levels but not with the anthocyanin content [53]. These diverse results may reflect the different methods used for sample preparation, e.g. in the latter study the authors dried the samples at 45°C which can result in the degradation of polyphenols, particularly anthocyanins [53].

### Artificial simplified phytocomplexes reveal synergy between different classes of metabolites

To investigate the *in vitro* antioxidant properties of the individual components of sweet cherry extracts in more detail, pure cyanidins, quercetins, caffeic acids and L-ascorbic acid were combined at the same concentrations found in three cultivars (Sandra Tardiva, Sandra and Grace Star) which were selected because they contain high, medium and low levels of these secondary metabolites, respectively. The resulting artificial simplified phytocomplexes provided a simplified representation of the antioxidant metabolome of each cultivar, although it was not possible to include the abundant hydroxycinnamic acid component coumaroyl quinic acid because it was not available as a pure compound. This approach allowed us to study the properties of the most abundant biologically active phytochemical classes in isolation, and to characterize their interactions, without the background noise of the rest of the sweet cherry metabolome. The advantage of this approach is that the phytocomplexes share the major properties of natural extracts but have a completely defined composition and can be manipulated to experimentally investigate the contributions of individual components. However, they only approximate the natural extracts and lack certain key components (e.g. procyanidins and the coumaroyl quinic acids) together with many identified and unidentified low-level metabolites, and thus cannot exactly reproduce the properties of natural extracts.

The *in vitro* antioxidant activity of the three artificial simplified phytocomplexes ranged from ~80% of the natural extract activity in the case of cultivar Sandra Tardiva to ~40% in the case of Sandra and Grace Star, suggesting that such phytocomplexes provide a valuable experimental model for some cultivars, specifically those where most of the natural antioxidant activity is represented by metabolites that have been included in the phytocomplexes. The missing antioxidant activities are therefore likely to reflect the contribution of coumaroyl quinic acid and lower-level phenolic compounds.

We compared the antioxidant activity of whole artificial simplified phytocomplexes with those specifically depleted for one class of molecules and with isolated metabolites. Remarkably, this experiment revealed that anthocyanins, quercetins and ascorbic acid, when mixed together at the same concentrations found in the Sandra Tardiva extracts, displayed strong synergistic antioxidant activity in the *in vitro* FRAP assay, but when the same components were mixed in the proportions found in the Sandra and Grace Star cultivars, the effects were additive rather than synergistic. Furthermore, only these three components showed synergy, whereas the hydroxycinnamic acids showed only additive effects. We cannot be certain that these synergistic effects among anthocyanins, quercetins and ascorbic acid are also present in the much more complex natural Sandra Tardiva extracts, but such an effect would help to explain the unanticipated strong correlation between antioxidant activity and the presence of quercetins, and the weaker correlation between antioxidant activity and the presence of hydroxycinnamic acids, despite the much lower abundance of quercetins in the extracts and the fact that both quercetins and hydroxycinnamic acids show similar levels of antioxidant activity when presented in isolation.

The consequences of synergistic interactions among antioxidants would mean that the properties of whole fruits cannot be determined accurately using *in vitro* assays based on isolated components. Furthermore, depending on the compositional context, low levels of some antioxidants could have a much higher impact than anticipated, as demonstrated by the relatively large effect of small amounts of ascorbic acid in Sandra Tardiva artificial phytocomplex compared to the relatively minor effect of the same quantity of ascorbic acid in the Sandra and Grace Star phytocomplexes. Finally, given that synergy in this study was predominantly seen in the Sandra Tardiva phytocomplex, representing a cultivar characterized by high levels of polyphenols, the difference in nutritional benefits between cultivars rich in polyphenols and those with lower levels could be much greater than predicted by composition alone.

These results were obtained using FRAP *in vitro* assay, that is based on the reduction of an artificial probe (ferric ions) that change colour when reduced by the tested antioxidant molecules [54]. This method is largely used for the simplicity of the experimental conditions [55]. However, the *in vivo* antioxidant properties of the investigated natural and artificial matrices cannot be simply deduced from the presented experiments, for various reasons. First, the used assays does not involve physiologically important oxidants such as the Reactive Oxygen Species generated by oxidative stress within the cells; second, in order to exert the antioxidant activity on humans the compounds have to reach the cells, which imply their bioavailability after the gastro intestinal tract transit.

Whether or not the observations above can be generalized from the phytocomplexes to the natural extracts, and from *in vitro* to *in vivo* conditions, synergistic and antagonistic effects among polyphenols have been reported before by several authors using authentic standards at one or a few selected concentrations, mixed at different molar or dose ratios, in binary, ternary and higher-order combinations. Additive, synergistic and/or antagonistic effects have been reported among flavonols (quercetin and quercetin-3-O-rutinoside) and hydroxycinnamic/hydroxybenzoic acids (gallic, rosmarinic and ferulloyl quinic acids) at two concentrations and in binary and ternary combinations [56], among flavonols (kaempferol, myricetin, quercetin and quercetin-3-O-glucoside), flavan-3-ols (catechin and epicatechin) and anthocyanins (pelargonidin, cyanidin, delphinidin, peonifdin and malvidin, all in the 3-O-glucoside form) in different molar ratios and in binary combinations [57], between the flavone calycosin and isoferulic acid at different dose ratios [58], among equimolar binary and ternary mixtures of quercetin, resveratrol and caffeic acid [59], and among flavonoids (quercetin and quercetin-3-O-rutinoside), flavan-3-ols (catechin and epicatechin) and resveratrol at one fixed concentration by combining up to five standard compounds, the later resulting in predominantly antagonistic rather than synergistic effects [60]. Synergistic antioxidant activity has been proposed to depend on the reciprocal ratio of the components with antioxidant properties [61] but is not clear how the phytochemicals in this study interact with each other at the molecular level and how these interactions are translated into antioxidant activity. The effects observed *in vitro* must be investigated in more detail and replicated using *in vivo* models.

## Conclusions

Our data show that the metabolic composition of sweet cherry extracts is mainly cultivar-dependent, whereas environmental factors that affect secondary metabolism play a less significant role. The *in vitro* antioxidant activity of sweet cherry extracts, measured by FRAP and ABTS assays, depends largely on the phenolic composition, and primarily on the anthocyanin content, both of which are influenced strongly by the cultivar. Moreover, strong synergistic interactions between the anthocyanins and quercetins/ascorbic acid were revealed by using an artificial simplified phytocomplex representing Sandra Tardiva composition. In order to gain insight into the real *in vivo* antioxidant activity of the natural and artificial cherry matrices, bioavailability investigation of cherry components and *in vivo* antioxidant assays must be performed.

## Supporting information

S1 File Supplementary figures. **Figure A. LC-MS base peak chromatograms and PCA score scatter plot showing different sweet cherry cultivars.** Three sweet cherry cultivars were selected and each trend can be compared with the corresponding chromatograms of the variety collected in a specific year. Each box shows the chromatographic trend of the three biological replicates. The PCA score scatter plot shows the clustering of the Burlat (green), Sandra Tardiva (blue) and Early Bigi (pink) samples. Circles indicate samples collected in 2014 and triangles indicate samples collected in 2015. Pink boxes indicate the Early Bigi samples collected in Sicily during 2015. **Figure B. Multivariate statistical analysis of primary and secondary metabolites.** A) PCA score scatter plot showing the clustering of specific cultivars depending on secondary metabolites. B) PCA loading plot showing the metabolites responsible for the sample clustering observed in the corresponding PCA score scatter plot (A). C) PCA score scatter plot showing the sample disposition based on the content of primary metabolites. Circles highlight the different collection years. D) PCA score and loading plots resulting from PCA-X analysis using primary metabolites as X variables. The plots highlight the vintage effect in nine cultivars. E) PCA score and loading plots resulting from PCA-X analysis using secondary metabolites as X variables for two specific varieties. **Figure C. Comparison of HPLC-DAD and LC-MS data.** A coloured heat map shows the percentage of specific metabolites among the different cultivars. Green indicates the lowest level and red the highest. Each value is the mean of the biological replicates spanning two collection years. Abbreviations: Cyanidin 3-O-glucoside and cyanidin 3-O-rutinoside, CyG+CyR. **Figure D. Heat map of LC-MS data reporting individual metabolite levels among the cultivars.** Values represent the peak areas. Green indicates the lowest level and red the highest. **Figure E. PCA score scatter plots showing sample clustering using secondary metabolites as the X variables.** The first plot (A) shows the sample disposition along the two principal components t1 and t2, the second (B) along the third (t3) and forth (t4) components. **Figure F. Predictive component tp versus Y for ABTS and FRAP data based on the targeted and untargeted metabolomics experiments.** PLS models were post-transformed in order to calculate the predictive components. (A) tp versus ABTS for targeted analysis; (B) tp versus log_10_(FRAP) for targeted analysis; (C) tp versus ABTS for untargeted analysis; (D) tp versus log_10_(FRAP) for untargeted analysis.
(PDF)

S1 Table Agronomic and climatic data for each sweet cherry cultivar. A) The orchard geographic coordinates with the corresponding cultivars, the harvest dates for the two growing seasons (2014 and 2015), the degrees Brix and pH values. B) Climatic details, expressed as rainfall (mm/month and days of rain/month) and temperature (°C), recovered from the four weather stations located near the orchards.
(XLSX)

S2 Table Composition of artificial simplified phytocomplexes. The precise quantity (mg/ml) of each specific metabolite is shown representing the Sandra Tardiva, Sandra and Grace Star extracts.
(XLS)

S3 Table Metabolic profiles of fruits representing 18 sweet cherry cultivars (2014 and 2015 vintages). Quantities were determined by NMR spectroscopy and HPLC-DAD for primary and secondary metabolites, respectively. Values are expressed as mg per 100 g fresh fruit and are means ± standard deviation (n = 3). Abbreviations: neochlorogenic acid, Neochlor; coumaroyl quinic acid, CoQ; chlorogenic acid, Chlor; cyanidin 3-O-glucoside and cyanidin 3-O-rutinoside, CyG+CyR; peonidin 3-O-rutinoside, PeR; quercetin 3-O-rutinoside, QR; quercetin 3-O-glucoside, QG; Ascorbic acid, Asc; valine, Val, threonine, Thr, alanine, Ala; asparagine, Aspn; malic acid, Mal; fumaric acid, Fum; fructose, Fruct; xylose, Xyl; glucose, Gluc.
(XLS)

S4 Table LC-MS data matrix including putatively identified and unidentified metabolites. The matrix shows the peak area of each detected signal and, for fragmented ions, the putative identification. Id: identifier; *m/z* (-) mass to charge ratio in negative ion mode; rt: retention time.
(XLSX)

S5 Table Stability selection output. Selected relevant metabolites from the analysis of the untargeted dataset by stability selection.
(XLSX)

S6 Table Linear regression output. Selected relevant metabolites from the simple linear regression analysis applied to the untargeted dataset.
(XLSX)
