# Supplementary material for: Multi-approach metabolomics analysis and artificial simplified phytocomplexes reveal cultivar-dependent synergy between polyphenols and ascorbic acid in fruits of the sweet cherry (Prunus avium L.)
Source: PLoS One. 2017 Jul 21;12(7):e0180889. doi: 10.1371/journal.pone.0180889 (PMC5521804; doi:10.1371/journal.pone.0180889)
Supplement: S1 File — Figure A. LC-MS base peak chromatograms and PCA score scatter plot showing different sweet cherry cultivars. Three sweet cherry cultivars were selected and each trend can be compared with the corresponding chromatograms of the variety collected in a specific year. Each box shows the chromatographic trend of the three biological replicates. The PCA score scatter plot shows the clustering of the Burlat (green), Sandra Tardiva (blue) and Early Bigi (pink) samples. Circles indicate samples collected in 2014 and triangles indicate samples collected in 2015. Pink boxes indicate the Early Bigi samples collected in Sicily during 2015. Figure B. Multivariate statistical analysis of primary and secondary metabolites. A) PCA score scatter plot showing the clustering of specific cultivars depending on secondary metabolites. B) PCA loading plot showing the metabolites responsible for the sample clustering observed in the corresponding PCA score scatter plot (A). C) PCA score scatter plot showing the sample disposition based on the content of primary metabolites. Circles highlight the different collection years. D) PCA score and loading plots resulting from PCA-X analysis using primary metabolites as X variables. The plots highlight the vintage effect in nine cultivars. E) PCA score and loading plots resulting from PCA-X analysis using secondary metabolites as X variables for two specific varieties. Figure C. Comparison of HPLC-DAD and LC-MS data. A coloured heat map shows the percentage of specific metabolites among the different cultivars. Green indicates the lowest level and red the highest. Each value is the mean of the biological replicates spanning two collection years. Abbreviations: Cyanidin 3-O-glucoside and cyanidin 3-O-rutinoside, CyG+CyR. Figure D. Heat map of LC-MS data reporting individual metabolite levels among the cultivars. Values represent the peak areas. Green indicates the lowest level and red the highest. Figure E. PCA score scatter plots showing sample clus [file pone.0180889.s001.pdf]

Multi-approach metabolomics analysis and artificial simplified phytocomplexes reveal cultivar-dependent synergy between polyphenols and ascorbic acid in fruits of the sweet cherry (*Prunus avium* L.)

Mauro Commisso[1¶], Martino Bianconi[1¶], Flavia Di Carlo[2], Stefania Poletti[1], Alessandra Bulgarini[1], Francesca Munari[1], Stefano Negri[1], Matteo Stocchero[3], Stefania Ceoldo[1], Linda Avesani[1], Michael Assfalg[1], Gianni Zoccatelli[1], Flavia Guzzo[1]*.

[1] Department of Biotechnology, University of Verona, Verona, Italy.

[2] Department of Biological, Chemical and Pharmaceutical Sciences and Technologies, University of Palermo, Palermo, Italy.

[3] Department of Women's and Children's Health, University of Padova, Padova, Italy.

*Corresponding author

E-mail: flavia.guzzo@univr.it

[¶]These authors contributed equally to this work.

S1_File including all supporting figures.

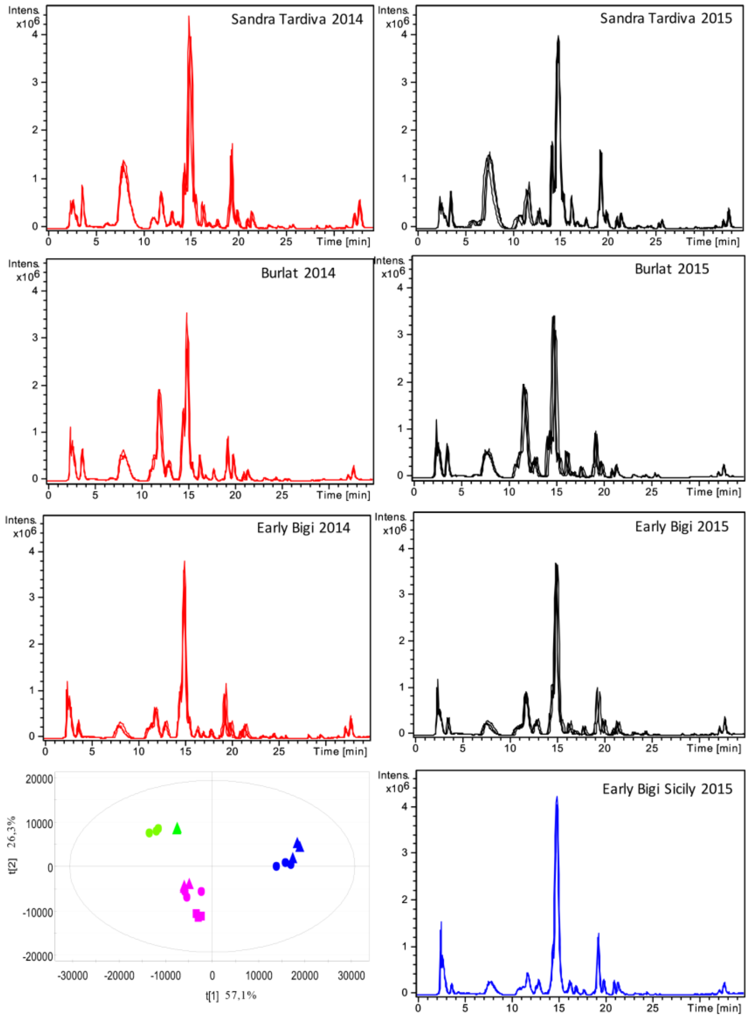

Figure A: LC-MS base peak chromatograms and PCA score scatter plot showing different sweet cherry cultivars. Three sweet cherry cultivars were selected and each trend can be compared with the corresponding chromatograms of the variety collected in a specific year. Each box shows the chromatographic trend of the three biological replicates. The PCA score scatter plot shows the clustering of the Burlat (green), Sandra Tardiva (blue) and Early Bigi (pink) samples. Circles indicate samples collected in 2014 and triangles indicate samples collected in 2015. Pink boxes indicate the Early Bigi samples collected in Sicily during 2015.

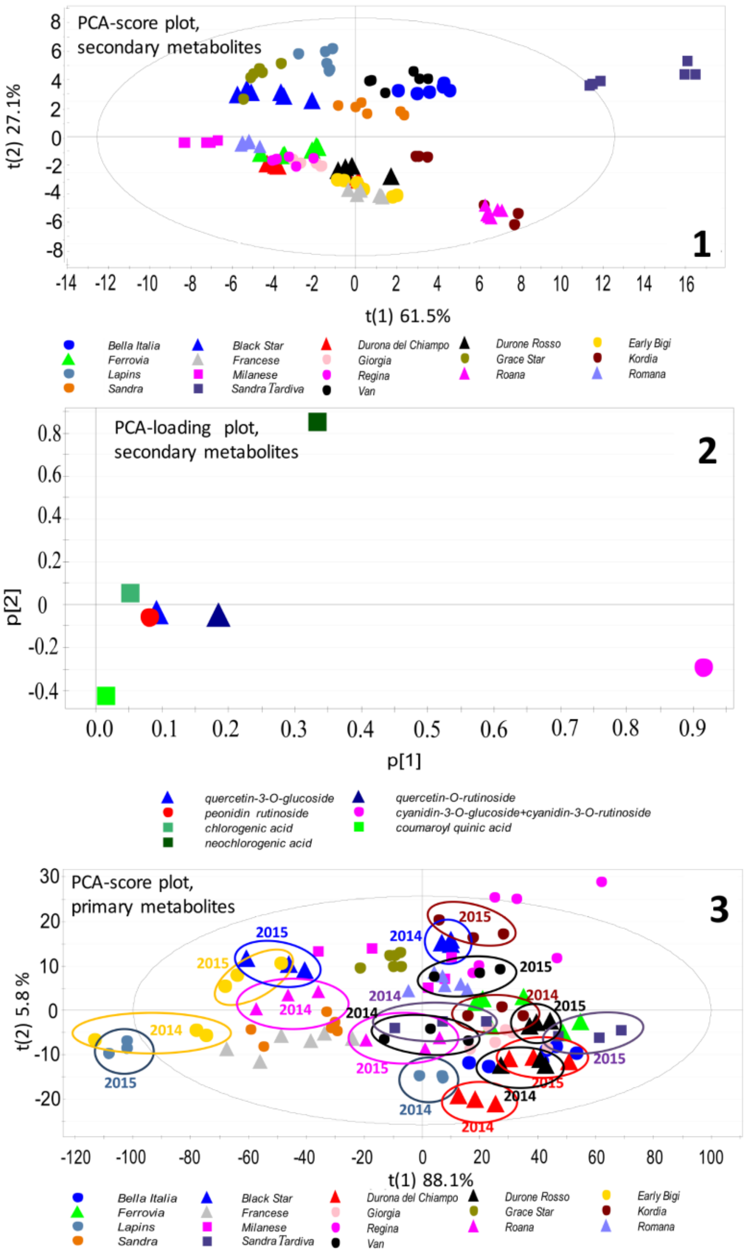
PCA-score plot,
secondary metabolites
1
t(2) 27.1%
t(1) 61.5%
Bella Italia
Ferrovia
Lapins
Sandra
Black Star
Francese
Milanese
Sandra Tardiva
Durona del Chiampo
Giorgia
Regina
Van
Durone Rosso
Grace Star
Roana
Early Bigi
Kordia
Romana
PCA-loading plot,
secondary metabolites
2
p[2]
p[1]
quercetin-3-O-glucoside
peonidin rutinoside
chlorogenic acid
neochlorogenic acid
quercetin-O-rutinoside
cyanidin-3-O-glucoside+cyanidin-3-O-rutinoside
coumaroyl quinic acid
PCA-score plot,
primary metabolites
3
t(2) 5.8 %
t(1) 88.1%
2014
2015
Bella Italia
Ferrovia
Lapins
Sandra
Black Star
Francese
Milanese
Sandra Tardiva
Durona del Chiampo
Giorgia
Regina
Van
Durone Rosso
Grace Star
Roana
Early Bigi
Kordia
Romana

PCA with primary metabolites as X variables

4

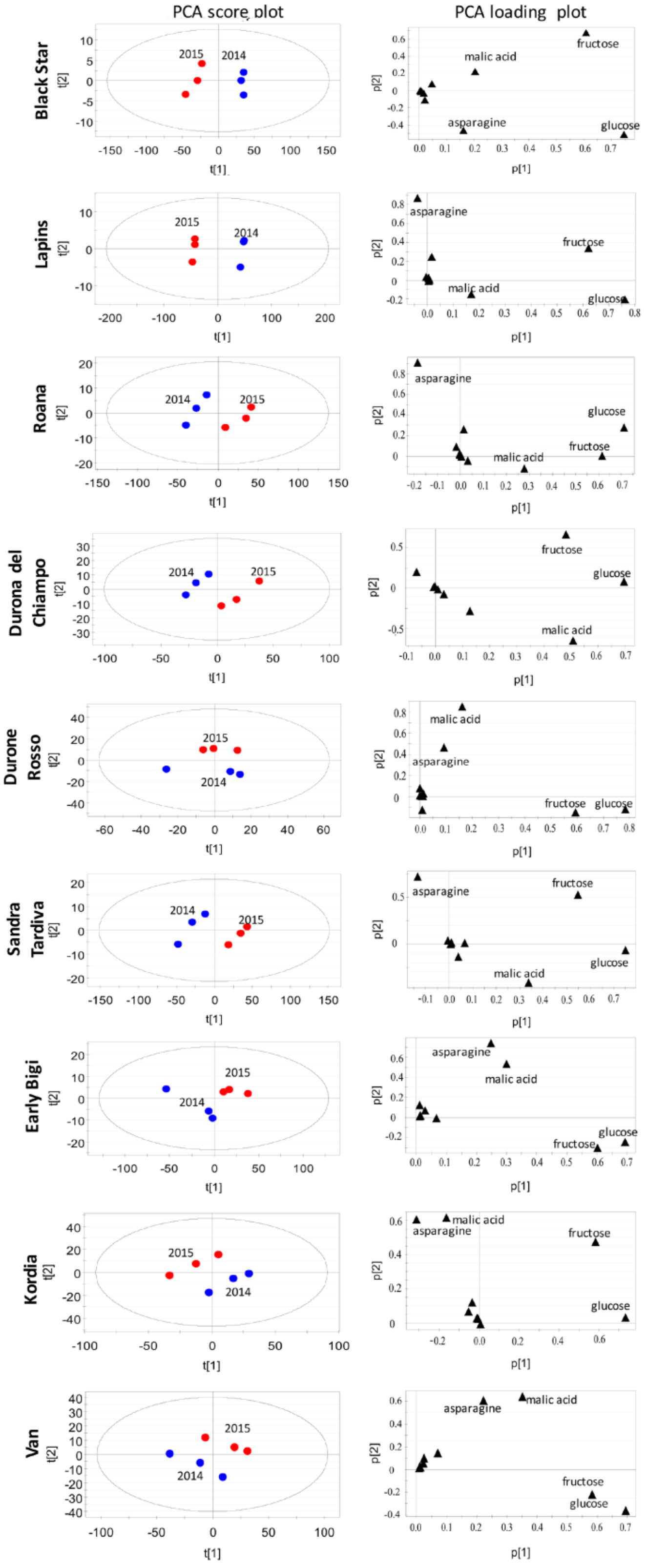

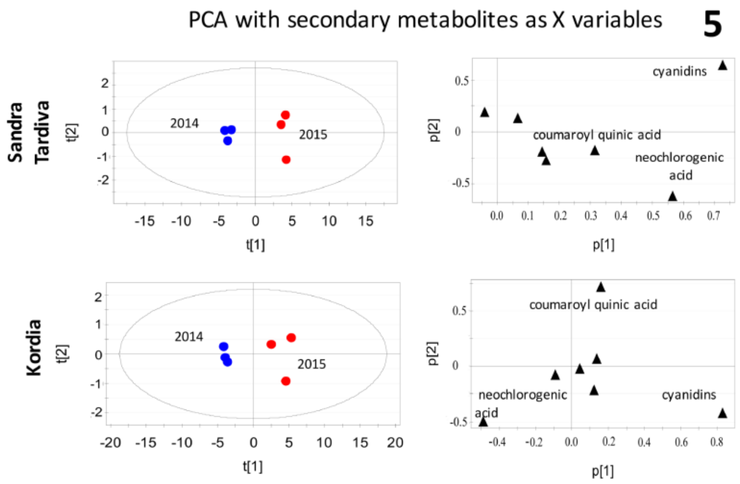

Figure B: Multivariate statistical analysis of primary and secondary metabolites. 1) PCA score scatter plot showing the clustering of specific cultivars depending on secondary metabolites. 2) PCA loading plot showing the metabolites responsible for the sample clustering observed in the corresponding PCA score scatter plot (1). 3) PCA score scatter plot showing the sample disposition based on the content of primary metabolites. Circles highlight the different collection years. 4) PCA score and loading plots resulting from PCA-X analysis using primary metabolites as X variables. The plots highlight the vintage effect in nine cultivars. 5) PCA score and loading plots resulting from PCA-X analysis using secondary metabolites as X variables for two specific varieties.

HPLC-DAD	Bella Italia	Black Star	Durona del Chiampo	Durone Rosso	Early Bigi	Ferrovia	Burlat	Giorgia	Grace Star
Neochlorogenic acid	73.9	43.2	18.7	23.4	11.7	27.3	24.5	25.2	52.9
Coumaroyl quinic acid	59.1	13.9	53.8	55.9	36.7	56.9	100.0	65.2	22.6
Chlorogenic acid	100.0	45.4	52.6	72.0	65.8	69.4	81.3	53.1	77.3
CyG+CyR	57.8	28.1	42.9	51.9	57.1	37.8	54.7	41.0	23.6
Peonidin-3-O-rutinoside	22.5	36.0	52.1	33.2	44.2	18.5	28.5	44.2	30.3
Quercetin-3-O-rutinoside	39.9	27.9	38.2	34.5	100.0	31.5	32.8	32.0	18.7
Quercetin-3-O-glucoside	44.1	-1.8	14.4	17.8	60.0	2.5	95.0	2.8	-8.3

HPLC-MS	Bella Italia	Black Star	Durona del Chiampo	Durone Rosso	Early Bigi	Ferrovia	Burlat	Giorgia	Grace Star
Neochlorogenic acid	66.6	40.4	36.9	46.3	19.5	37.6	26.8	36.1	79.4
Coumaroyl quinic acid	58.6	15.8	60.3	59.5	41.9	62.4	100.0	57.6	24.6
Chlorogenic acid	100.0	54.9	59.8	76.9	77.3	86.2	93.3	57.0	85.3
CyG+CyR	83.5	59.2	70.8	81.6	84.2	69.6	78.5	65.2	51.2
Peonidin-3-O-rutinoside	17.3	47.7	54.8	33.9	44.0	20.7	23.7	43.2	38.2
Quercetin-3-O-rutinoside	69.1	44.5	61.6	58.6	60.9	52.7	54.2	51.6	34.5
Quercetin-3-O-glucoside	61.6	14.6	29.2	36.4	75.1	21.8	100.2	19.5	10.3

HPLC-DAD	Kordia	Lapins	Milanese	Regina	Roana	Romana	Sandra	Sandra Tardiva	Van
Neochlorogenic acid	33.0	68.3	28.5	28.1	23.1	35.5	58.6	100.0	65.6
Coumaroyl quinic acid	70.8	21.6	55.7	80.9	76.6	95.5	60.0	42.7	18.9
Chlorogenic acid	60.0	75.5	61.7	69.2	69.9	81.0	56.6	87.8	73.6
CyG+CyR	74.5	35.4	16.7	35.3	83.3	23.9	47.6	100.0	52.2
Peonidin-3-O-rutinoside	100.0	36.8	5.4	20.2	49.5	26.3	23.1	40.9	58.0
Quercetin-3-O-rutinoside	47.4	21.5	21.8	31.5	58.0	28.0	33.4	77.9	37.3
Quercetin-3-O-glucoside	19.5	-13.1	-15.8	-6.7	72.8	4.6	100.0	91.5	34.9

HPLC-MS	Kordia	Lapins	Milanese	Regina	Roana	Romana	Sandra	Sandra Tardiva	Van
Neochlorogenic acid	35.9	73.5	34.5	38.7	18.9	55.8	74.5	100.0	65.6
Coumaroyl quinic acid	62.1	21.5	58.1	76.3	69.7	87.9	59.3	32.4	21.5
Chlorogenic acid	65.2	91.2	69.7	79.5	75.3	87.3	76.1	79.1	83.5
CyG+CyR	99.2	62.8	40.1	63.1	97.4	52.3	75.3	100.0	80.4
Peonidin-3-O-rutinoside	100.0	44.3	11.0	21.9	43.9	32.4	19.9	21.8	68.4
Quercetin-3-O-rutinoside	76.5	42.5	40.6	53.3	90.1	48.8	55.3	100.0	59.8
Quercetin-3-O-glucoside	40.1	11.7	11.5	14.8	77.1	24.7	100.0	79.7	40.9

Figure C: Comparison of HPLC-DAD and LC-MS data. A coloured heat map shows the percentage of specific metabolites among the different cultivars. Green indicates the lowest level and red the highest. Each value is the mean of the biological replicates spanning two collection years. Abbreviations: Cyanidin 3-O-glucoside and cyanidin 3-O-rutinoside, CyG+CyR.

	Bella Italia	Black Star	Durona del Chiampo	Durone Rosso	Early Bigi	Ferrovia	Burlat	Giorgia	Grace Star	Kordia	Lapins	Milanese	Regina	Roana	Romana	Sandra Tardiva	Sandra	Van	
Neochlorogenic acid	47,406,224	28,723,119	26,243,009	32,926,828	13,892,567	26,731,220	19,063,754	25,683,842	56,467,034	25,522,747	52,311,873	24,580,575	27,528,213	13,468,935	39,723,318	71,160,087	53,005,550	46,672,584	Hydroxycinnamic acids and hydroxybenzoic acids
Chlorogenic acid	9,508,501	5,219,048	5,690,573	7,309,698	7,347,772	8,200,915	8,870,869	5,419,591	8,109,773	6,203,421	8,673,585	6,629,446	7,559,351	7,158,821	8,302,058	7,517,525	7,236,979	7,943,220	
Caffeoyl quinic acid adduct	4,945,154	1,391,043	1,401,742	2,901,327	2,875,618	3,302,075	4,060,155	1,491,207	3,301,524	2,030,707	3,785,375	2,460,323	3,122,367	3,133,133	3,934,885	3,350,895	2,583,289	3,154,728	
Caffeoyl quinic acid methyl derivative	3,985,494	1,750,081	2,673,335	2,958,214	2,864,738	3,055,752	3,428,084	2,056,242	3,145,180	1,861,355	3,537,741	2,701,768	2,607,065	2,952,457	3,339,550	2,803,879	2,516,916	2,462,612	
Caffeoyl quinic acid 1	2,559,193	3,395,816	1,049,387	1,194,643	526,315	2,044,901	1,915,626	1,110,034	2,323,201	1,146,965	2,590,038	1,826,766	1,632,225	636,594	2,214,790	1,872,808	2,452,219	2,778,313	
Caffeoyl quinic acid hexose 2	772,337	1,653,460	1,569,584	823,556	112,097	1,073,147	229,436	1,952,257	2,016,578	1,209,351	3,167,917	1,212,926	1,873,066	248,134	886,357	5,325,694	333,722	1,237,275	
Caffeoyl quinic acid hexose 1	1,899,533	1,491,724	329,977	644,804	96,611	579,226	340,896	1,262,614	780,304	1,512,227	1,772,645	1,411,763	817,555	228,108	965,215	2,828,796	222,195	2,089,141	
Caffeoyl quinic acid 4	1,040,433	927,305	954,313	1,115,347	880,560	1,189,879	1,193,600	756,530	1,089,163	801,241	937,666	892,133	1,086,140	734,397	1,019,008	768,318	812,474	828,394	
Caffeoyl quinic acid 2 adduct	1,221,907	440,014	423,132	811,071	792,462	874,441	998,920	473,625	829,759	620,594	906,787	696,122	1,002,944	797,984	982,481	904,971	740,715	885,311	
Caffeoyl hexose	773,193	1,052,602	566,160	500,225	257,626	547,917	230,661	801,745	642,569	882,226	1,026,289	666,545	1,006,218	176,222	566,332	1,671,333	304,739	859,596	
Caffeoyl quinic hexose 3	829,530	511,060	182,755	352,314	74,808	511,555	153,527	575,598	216,175	856,058	334,925	854,390	666,504	138,990	920,762	1,126,101	172,602	1,342,507	
Caffeoyl quinic acid adduct	1,789,879	202,856	133,286	247,363	69,700	216,094	284,587	161,580	187,637	262,238	1,385,621	398,654	143,220	160,266	389,605	1,549,207	533,184	845,594	
Caffeoyl quinic acid derivative	202,186	310,791	235,485	127,698	51,398	341,396	72,466	186,948	227,418	128,452	309,886	167,890	160,365	107,315	192,783	378,194	167,178	157,555	
Feruloyl quinic acid	15,933,412	13,344,331	5,760,032	5,939,809	2,646,075	7,080,532	5,269,694	6,000,756	15,833,725	5,955,905	19,555,085	7,764,793	6,587,000	5,123,953	8,425,195	17,177,277	13,872,653	14,448,516	
Dicaffeoyl quinic acid	6,097,330	4,728,987	2,322,202	5,211,030	5,565,189	5,492,371	4,774,544	3,619,098	5,841,632	4,457,724	6,629,527	3,143,865	4,440,977	3,108,406	4,282,448	6,106,026	3,495,020	6,582,202	
Coumaroyl quinic acid 1	42,742,406	11,501,763	43,955,400	43,374,405	30,547,619	45,529,448	72,906,277	41,978,523	17,918,787	45,253,819	15,698,138	42,386,260	55,662,780	50,805,898	64,053,233	23,617,255	43,215,483	15,663,096	
Coumaroyl quinic acid 1 adduct	2,728,087	260,669	2,147,137	2,638,363	1,329,804	2,852,746	7,168,426	2,920,454	473,381	3,244,271	528,687	2,777,770	4,387,859	3,677,900	5,321,318	1,215,324	3,024,409	605,271	
Coumaroyl caffeoyl quinic acid	1,860,261	531,876	1,001,783	1,785,717	1,522,558	1,528,402	1,628,016	1,040,163	808,940	1,359,098	897,830	913,881	1,467,645	1,114,974	1,551,281	1,657,302	1,213,802	827,593	
Coumaroyl quinic acid 3	1,074,708	296,833	1,253,452	1,150,141	673,477	1,369,310	1,426,323	1,010,645	372,263	1,174,369	444,743	1,812,355	1,532,385	1,606,996	1,761,454	796,363	663,912	331,111	
Coumaroyl quinic acid 2	855,195	282,894	1,647,342	1,508,085	578,836	1,798,618	1,036,083	1,297,099	271,591	1,738,744	299,013	1,621,092	1,837,980	1,698,539	1,663,687	426,185	292,388	349,223	
Coumaroyl quinic acid adduct	516,357	365,624	436,295	695,134	694,565	577,905	552,540	428,532	242,735	550,796	392,328	363,846	727,090	1,321,492	336,102	796,747	495,573	706,830	
Syringic acid hexose derivative	800,543	121,368	775,667	766,846	741,050	839,080	560,312	342,420	753,603	1,200,552	685,485	743,950	1,139,325	810,323	449,936	884,153	71,377	643,503	
Hydroxybenzoic acid derivative, chloride adduct	345,079	1,079,905	444,467	1,037,238	273,419	815,067	1,881,549	1,192,832	471,891	917,319	713,601	522,760	135,126	1,171,075	562,105	822,411	1,260,455	516,393	
Hydroxybenzoic acid hexose, chloride adduct	262,783	225,689	318,635	209,728	127,822	293,823	120,788	243,116	235,328	157,725	416,096	200,829	296,286	80,766	295,769	375,045	94,821	140,220	
Cyanidin-3-O-rutinoside	93,810,314	75,642,866	87,886,639	99,795,013	91,338,006	88,187,148	79,675,298	82,643,723	65,773,387	122,023,101	81,260,116	51,562,806	82,075,081	113,329,378	64,080,196	118,393,135	77,831,498	97,913,930	Anthocyanins
Cyanidin-3-O-rutinoside hydrate	20,190,970	12,241,288	15,430,278	18,983,237	18,205,163	15,286,892	13,786,703	15,461,023	10,342,036	27,951,191	14,154,202	8,607,377	14,490,791	24,069,008	11,523,611	27,351,434	13,068,067	19,685,572	
Cyanidin-3-O-rutinoside, isotope	6,055,393	1,097,867	1,572,824	2,716,978	4,619,756	2,138,315	2,026,828	1,613,869	2,253,644	3,529,199	1,607,295	2,223,115	1,429,945	4,695,860	3,595,818	4,346,373	3,927,536	3,069,121	
Cyanidin-3-O-rutinoside hydrate, chloride adduct	731,353	1,353,590	1,504,834	1,543,765	1,173,362	1,463,729	1,418,665	1,411,629	1,018,341	1,610,184	1,295,156	673,157	1,325,471	1,549,078	839,371	1,596,617	1,521,338	1,107,861	
Cyanidin-3-O-rutinoside hydrate, chloride adduct	642,789	1,158,361	1,210,883	1,457,168	933,368	1,131,781	1,098,640	1,151,117	786,242	1,323,791	1,230,771	382,540	1,131,045	1,516,566	540,361	1,014,752	1,034,413	968,117	
Cyanidin-3-O-rutinoside hydrate, chloride adduct	1,866,413	3,389,129	3,572,269	3,809,342	2,840,650	4,146,325	3,636,338	3,245,838	2,686,720	3,949,068	3,716,701	1,566,634	3,663,009	3,681,660	1,653,249	4,041,052	3,061,182	2,860,550	
Cyanidin-3-O-rutinoside hydrate, chloride adduct	237,940	410,682	433,371	423,560	283,005	446,594	361,968	378,621	394,226	431,257	476,973	219,110	466,877	468,753	227,862	336,298	443,932	407,434	
Pelargonidin-3-O-rutinoside	426,572	215,810	277,100	470,442	246,437	332,799	242,165	214,090	156,054	520,725	317,759	116,526	385,612	468,928	172,411	441,514	174,514	380,049	
Peonidin-3-O-rutinoside (*trans*)	1,804,001	4,975,495	5,718,776	3,542,182	4,597,065	2,161,634	2,469,303	4,511,957	3,989,264	10,439,296	4,622,911	1,146,127	2,282,330	4,581,459	3,387,074	2,276,735	2,075,126	7,145,372	
Peonidin-3-O-rutinoside, chloride adduct (*trans*)	626,219	1,616,763	2,082,433	1,171,772	1,530,996	735,250	783,488	1,649,265	1,214,611	3,857,945	1,426,528	310,771	749,433	1,403,897	1,082,934	635,882	672,739	2,239,668	
Cyanidin-3-O-glucoside	19,589,400	4,703,856	8,200,232	10,948,077	22,967,232	6,337,824	26,945,033	5,824,245	3,758,763	12,601,481	4,026,041	2,917,369	3,594,132	18,875,119	6,880,070	17,375,236	24,408,551	11,282,253	
Cyanidin-3-O-glucoside hydrate	5,835,392	813,536	1,642,870	2,441,341	6,118,367	1,057,547	7,024,402	1,154,350	642,815	3,225,810	669,575	515,890	669,278	4,970,359	1,527,698	5,146,882	6,326,468	2,367,205	
Cyanidin-3-glucoside, chloride adduct	1,053,986	560,055	924,980	985,001	2,057,263	830,998	3,559,985	701,660	485,841	1,080,076	507,776	167,645	410,437	1,726,125	549,938	1,577,257	2,595,146	1,344,405	
Cyanidin-3-glucoside, chloride adduct	483,576	309,269	567,884	550,124	961,540	453,188	1,810,761	424,365	288,193	479,843	292,381	133,965	249,093	887,310	312,738	804,248	1,370,946	540,877	
Cyanidin-3-glucoside, chloride adduct	488,035	157,847	474,447	484,423	844,173	437,570	1,289,067	323,736	205,418	557,700	177,380	83,584	210,612	731,406	165,500	736,445	1,121,076	503,299	
Quercetin-3-O-rutinoside	20,685,389	13,322,551	18,415,001	17,537,371	18,208,505	15,760,137	16,199,226	15,422,049	10,314,424	22,890,713	12,703,587	12,145,254	15,959,054	26,950,919	14,593,686	29,914,611	16,532,275	17,896,783	Flavonoids
Quercetin-3-O-glucoside	4,760,838	1,131,088	2,260,405	2,812,414	5,807,451	1,682,201	7,740,240	1,509,120	796,102	3,100,432	903,489	890,148	1,143,131	5,954,853	1,907,726	6,159,595	7,728,381	3,163,885	
Quercetin-3-O-rutinoside-O-hexoside	1,928,854	1,445,245	1,411,465	1,804,689	1,541,498	1,799,853	1,443,279	1,184,890	964,099	1,335,179	1,357,980	1,003,815	1,744,666	1,539,001	1,193,203	1,446,690	1,705,867	1,168,363	
Kaempferol-3-O-rutinoside	5,173,523	3,172,020	3,745,903	3,674,589	2,090,281	4,439,813	2,184,998	2,840,273	2,138,086	2,807,080	1,922,584	4,989,778	5,002,001	2,954,492	5,596,723	2,750,410	2,551,016	2,500,596	
Kaempferol-3-O-hexoside	890,050	167,807	279,601	326,170	272,873	254,619	170,027	286,036	120,869	425,148	124,319	542,393	612,756	628,768	575,875	652,588	526,522	122,105	
Trihydroxyflavon-O-hexoside, formic acid adduct	4,381,142	5,749,197	1,693,444	2,163,130	1,812,913	1,955,809	676,285	3,839,138	3,371,989	3,913,355	4,889,407	3,309,512	5,164,422	5,476,871	2,424,125	6,897,558	1,308,147	3,394,867	
Trihydroxyflavon-O-hexoside, chloride adduct	1,941,946	10,187,132	2,249,745	2,045,714	1,670,945	2,763,463	956,252	4,098,340	4,957,098	3,705,299	7,991,781	2,430,789	5,997,862	4,039,764	1,113,579	7,103,911	1,395,745	2,496,067	
Trihydroxyflavon-O-hexoside, chloride adduct	445,702	3,022,700	489,479	588,615	472,249	816,012	232,104	1,166,891	1,418,446	1,160,373	2,502,905	669,072	1,881,294	1,139,719	319,947	2,199,108	454,571	843,227	
Trihydroxyflavon-O-pentoside, chloride adduct	660,059	2,677,747	1,088,260	769,360	603,945	905,938	506,344	591,125	847,836	813,155	1,436,341	1,282,126	1,432,056	627,794	575,542	723,797	384,343	630,363	
Trihydroxyflavon-O-hexoside, chloride adduct	371,515	969,270	314,874	368,477	212,421	407,295	156,584	634,175	676,070	518,090	1,053,059	315,062	790,558	612,155	111,722	1,015,702	257,012	365,202	
Trihydroxyflavon-O-pentoside, chloride adduct	269,342	911,001	378,863	247,629	190,357	303,228	178,051	209,090	272,373	286,973	485,219	420,222	412,857	189,044	198,649	243,820	126,620	235,721	
Trihydroxyflavon-O-hexoside, chloride adduct	94,934	472,038	97,995	59,962	51,062	118,928	80,248	167,628	269,085	233,041	269,744	171,348	202,377	200,753	83,826	274,711	86,836	93,773	
Dihydroxyflavon-O-hexoside, chloride adduct	599,587	718,860	416,310	625,276	989,114	729,226	932,044	442,348	561,455	675,833	811,771	376,542	845,517	516,620	568,462	582,220	512,018	581,257	
Dihydroxyflavon-O-hexoside, formic acid adduct	847,227	425,092	251,527	504,765	1,169,787	407,258	557,782	245,415	347,293	654,707	464,143	361,227	566,047	364,383	611,199	448,435	345,192	534,307	
Epicatechin	9,619,485	2,739,489	3,575,473	5,868,552	6,089,659	5,913,858	6,764,894	6,648,067	5,673,452	6,813,807	5,947,810	4,081,236	8,968,577	5,804,826	7,289,242	12,423,516	4,530,383	4,211,320	Procyanidins and flavan-3-ols
Epicatechin, chloride adduct	1,863,477	1,492,762	1,747,230	2,306,144	2,053,386	2,815,017	3,279,857	2,930,698	2,608,717	2,207,984	2,799,875	1,384,727	4,033,956	1,965,007	1,968,337	3,734,581	2,014,994	1,420,136	
Epicatechin, chloride adduct	687,331	527,463	624,771	841,994	776,636	1,000,876	1,168,194	969,914	890,257	789,196	927,504	534,245	1,232,130	684,937	729,680	1,228,143	673,357	447,271	
Catechin	4,196,801	1,803,903	1,101,903	2,795,928	5,007,283	2,483,860	6,494,686	1,360,375	2,244,738	4,592,846	1,687,855	1,954,476	4,068,078	4,792,856	3,332,728	2,179,534	5,848,338	1,554,650	
Catechin, chloride adduct	1,064,684	890,945	682,108	1,094,075	1,617,219	1,135,618	2,739,160	871,116	1,123,595	1,647,060	832,692	724,623	1,713,560	1,591,172	958,276	690,988	2,183,820	632,137	
Catechin/epicatechin derivative	1,020,055	684,101	1,104,603	869,044	504,656	1,014,822	857,160	1,329,393	436,413	1,191,301	491,748	914,030	2,073,641	1,387,441	998,863	2,030,997	1,598,841	234,414	
Procyanidin P2	12,766,581	3,749,752	9,300,346	8,819,612	7,100,755	10,144,182	10,020,914	13,677,142	10,802,585	11,810,038	11,629,456	8,355,604	18,548,083	7,422,089	12,952,189	25,352,469	4,281,042	6,443,514	
Procyanidin P2	9,700,682	3,686,622	4,041,274	6,455,195	7,261,019	5,789,158	9,585,459	3,490,776	7,001,802	11,954,128	6,604,300	4,116,661	10,398,911	7,354,473	6,765,422	10,062,676	8,105,071	4,257,088	
Procyanidin P2	1,790,975	625,592	865,994	1,056,688	1,265,143	1,059,229	1,792,281	787,406	1,037,956	1,699,021	963,977	909,975	1,715,436	1,276,428	1,482,729	1,733,070	1,320,073	855,723	
Procyanidin P2	1,762,471	466,625	925,046	1,010,265	870,665	1,024,742	1,162,639	1,397,989	1,096,639	1,221,420	1,135,371	868,164	1,867,899	767,803	1,528,323	3,801,126	645,097	776,140	
Procyanidin P3	4,635,452	739,350	2,210,786	2,346,409	2,238,509	2,542,090	3,638,576	3,039,781	2,346,396	2,954,992	2,408,443	2,067,352	4,542,667	2,035,001	3,744,421	9,731,910	1,615,369	1,781,512	
Procyanidin P3	1,513,569	348,935	511,747	916,956	1,250,363	809,062	1,817,436	429,078	665,349	1,666,756	493,430	735,772	1,490,235	995,304	1,347,459	1,429,944	1,262,696	633,600	
Procyanidin P4	922,389	82,479	624,672	406,367	470,991	460,150	718,437	680,694	474,575	553,483	509,765	493,052	845,171	429,875	790,091	2,125,020	273,745	413,658	
Catechin, chloride adduct	348,879	424,425	125,790	428,489	544,775	422,322	780,052	246,097	313,448	547,268	320,574	268,095	544,243	506,065	319,693	279,872	738,660	174,619	

Figure D: Heat map of LC-MS data reporting individual metabolite levels among the cultivars.

Values represent the peak areas. Green indicates the lowest level and red the highest.

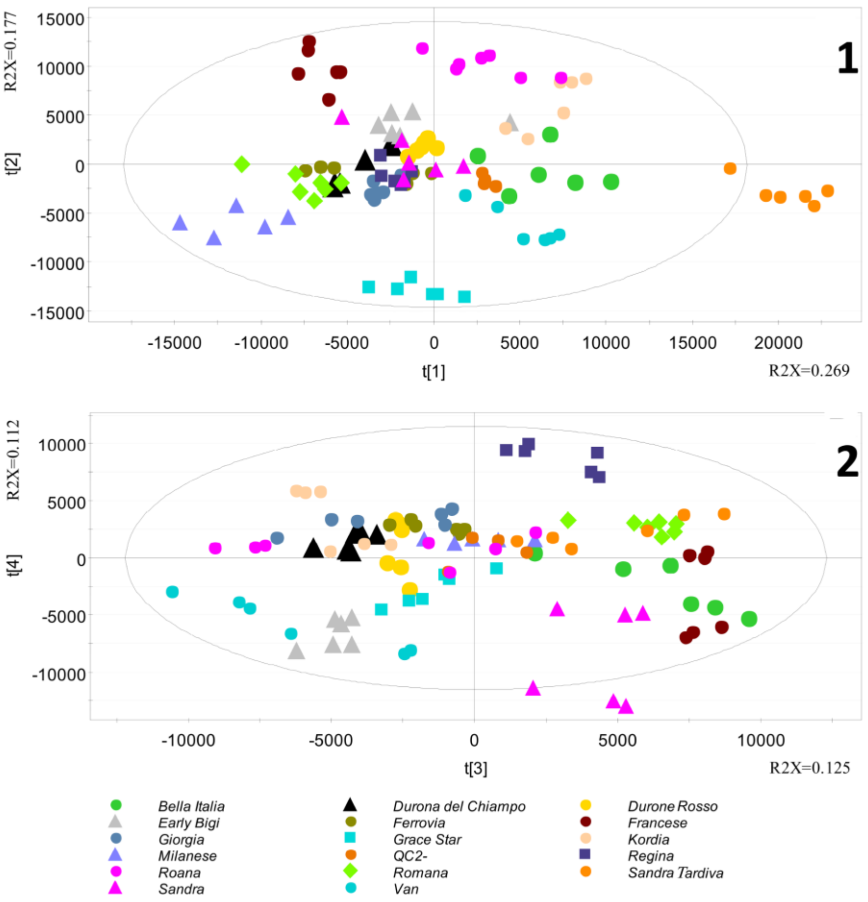

Figure E: PCA score scatter plots showing sample clustering using secondary metabolites as the X variables. The first plot (1) shows the sample disposition along the two principal components t1 and t2, the second (2) along the third (t3) and forth (t4) components.

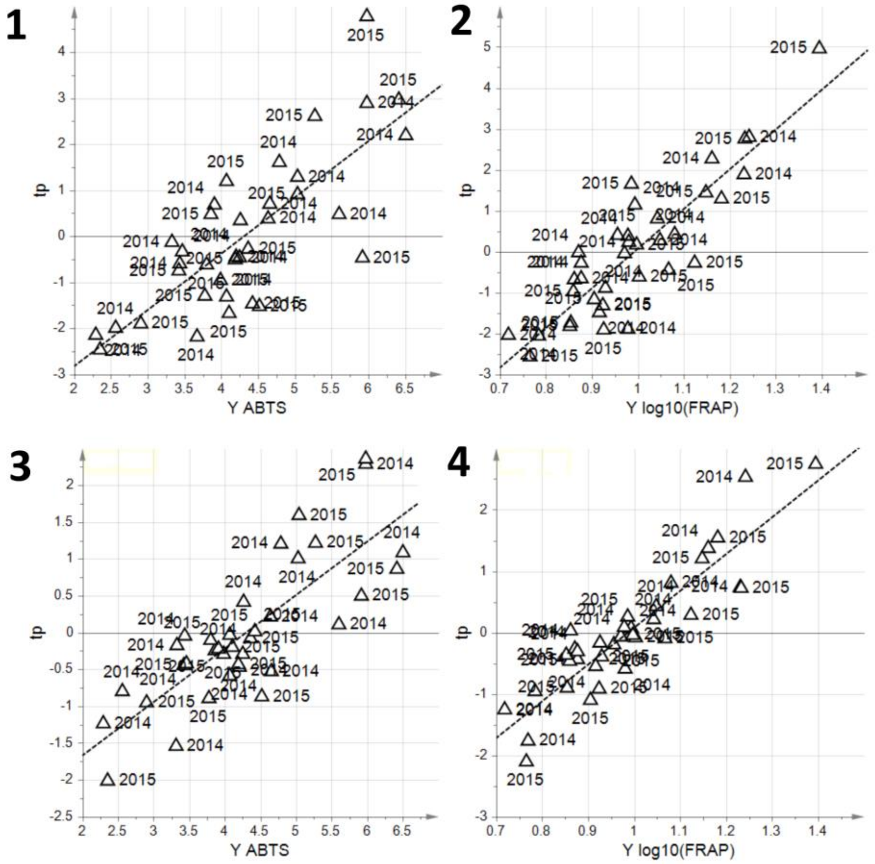

Figure F: Predictive component tp versus Y for ABTS and FRAP data based on the targeted and untargeted metabolomics experiments. PLS models were post-transformed in order to calculate the predictive components (Stocchero and Paris, 2016). (1) tp versus ABTS for targeted analysis; (2) tp versus $\log_{10}$(FRAP) for targeted analysis; (3) tp versus ABTS for untargeted analysis; (4) tp versus $\log_{10}$(FRAP) for untargeted analysis.

Literature cited

Stocchero M, Paris D. Post-transformation of PLS2 (ptPLS2) by orthogonal matrix: a new approach for generating predictive and orthogonal latent variables. J. Chemometrics. 2016;30: 242–251
